# Virus Satellites Drive Viral Evolution and Ecology

**DOI:** 10.1371/journal.pgen.1005609

**Published:** 2015-10-23

**Authors:** Belén Frígols, Nuria Quiles-Puchalt, Ignacio Mir-Sanchis, Jorge Donderis, Santiago F. Elena, Angus Buckling, Richard P. Novick, Alberto Marina, José R. Penadés

**Affiliations:** 1 Universidad Cardenal Herrera CEU, Moncada, Spain; 2 Institute of Infection, Immunity and Inflammation, College of Medical, Veterinary and Life Sciences, University of Glasgow, Glasgow, United Kingdom; 3 Instituto de Biomedicina de Valencia (CSIC), Valencia, Spain; 4 Instituto de Biología Molecular y Celular de Plantas (CSIC-UPV), Valencia, Spain; 5 Santa Fe Institute, Santa Fe, New Mexico, United States of America; 6 Department of Biosciences, Center for Ecology and Conservation, School of Biosciences, University of Exeter, Cornwall Campus, Penryn, United Kingdom; 7 Skirball Institute Program in Molecular Pathogenesis and Departments of Microbiology and Medicine, New York University Medical Center, New York, New York, United States of America; Fred Hutchinson Cancer Research Center, UNITED STATES

## Abstract

Virus satellites are widespread subcellular entities, present both in eukaryotic and in prokaryotic cells. Their *modus vivendi* involves parasitism of the life cycle of their inducing helper viruses, which assures their transmission to a new host. However, the evolutionary and ecological implications of satellites on helper viruses remain unclear. Here, using staphylococcal pathogenicity islands (SaPIs) as a model of virus satellites, we experimentally show that helper viruses rapidly evolve resistance to their virus satellites, preventing SaPI proliferation, and SaPIs in turn can readily evolve to overcome phage resistance. Genomic analyses of both these experimentally evolved strains as well as naturally occurring bacteriophages suggest that the SaPIs drive the coexistence of multiple alleles of the phage-coded SaPI inducing genes, as well as sometimes selecting for the absence of the SaPI depressing genes. We report similar (accidental) evolution of resistance to SaPIs in laboratory phages used for *Staphylococcus aureus* typing and also obtain the same qualitative results in both experimental evolution and phylogenetic studies of *Enterococcus faecalis* phages and their satellites viruses. In summary, our results suggest that helper and satellite viruses undergo rapid coevolution, which is likely to play a key role in the evolution and ecology of the viruses as well as their prokaryotic hosts.

## Author Summary

Satellites are defined as viruses that have a life cycle dependent on a helper virus. Thus, they can be considered as parasites of parasites. In addition to their fascinating life cycle, these widespread infectious elements, present both in eukaryotic and prokaryotic cells, have a dramatic role in virulence by controlling the symptoms induced by their eukaryotic helper viruses or by encoding key bacterial virulence genes. While satellites can play an important role in the ecology of the viruses they parasitise, the evolutionary impact on their helper viruses is unclear. Here we show that staphylococcal pathogenicity islands (SaPIs), an example of a virus satellite, are a major selective force on the viruses (bacteriophages) they parasitise. Using both bioinformatic and experimental evolution data we have been able to confirm that pathogenicity islands are a major selective pressure enhancing the diversity of both genes and gene content in *Staphylococcus aureus* phages. Since SaPIs exploit the life cycle of their helper phages to enable their rapid replication and promiscuous spread, these strategies are mechanisms that reduce SaPI interference, thus facilitating the infectivity and dissemination of the helper phages in nature.

## Introduction

Satellites are defined as viruses which have a life cycle dependent on a helper virus, but lack extensive nucleotide sequence homology to the helper virus and are dispensable for helper virus proliferation [1–4]. These infectious elements, present both in eukaryotic and prokaryotic cells, have far-reaching consequences. First, they can play a major role in the population dynamics of viruses and their hosts, with satellite viruses able to greatly limit the proliferation of their helper viruses. For example, the presence of *Staphylococcus aureus* pathogenicity islands (SaPIs), a type of satellite virus, reduces phage proliferation [5,6]. Given the crucial role of viruses in shaping microbial communities [7], satellite viruses may themselves be a key driver of microbial community structure and function. Second, satellite viruses can have a dramatic role in virulence by controlling the symptoms induced by their helper viruses or by encoding relevant virulence genes. For example, Hepatitis B virus (HBV) is a major health problem of global impact. Among the HBV chronically infected patients, many are co-infected with the Hepatitis delta virus (HDV), a satellite virus that needs the HBV for propagation. HDV is the smallest virus known to infect humans and is clinically relevant because it causes a fulminant hepatitis or a more rapid progression of liver disease in the setting of chronic HBV infection [8]. Satellite prokaryotic viruses (satellite phages) are also relevant both in the virulence and in the emergence of novel bacterial pathogens. In addition to the SaPIs, which have a relevant role in bacterial evolution and pathogenesis by encoding relevant virulence factors [9,10], *Vibrio cholerae* phage satellites control not only the expression of the clinically relevant CTX phage-coded cholera toxin, but also the transmission of their helper CTX phage [11,12].

Interactions between hosts and their parasites frequently result in antagonistic coevolution, with host evolving defence and parasites evolving counter defence [13]. Given that satellite viruses typically have negative consequences for their helper viruses, while the satellite viruses require ‘susceptible” viruses for their proliferation, antagonistic coevolution is a feasible outcome. Antagonistic coevolution can have major impacts on the ecology and evolution of viruses and their hosts. Specifically, the degree of resistance of helpers to their satellites will determine the spread of both helper and satellite viruses between their prokaryotic or eukaryotic hosts, which in turn affects host population dynamics and evolutionary trajectories. While the existence of satellite viruses clearly shows adaptation of satellites to helper viruses, it is currently unclear if satellite viruses drive significant evolutionary change in helper virus resistance and, if so, whether satellite viruses in turn evolve to overcome helper virus resistance. Here we address these questions for the interaction between the SaPIs and their inducing phages.

The SaPIs are the prototypical members of a widespread family of highly mobile pathogenicity islands, the PICIs (phage-inducible chromosomal islands), that exploit the life cycle of their helper phages with elegant precision to enable their rapid replication and promiscuous spread [4,10]. In the absence of helper phage lytic growth, the island is maintained in a quiescent prophage-like state by a global repressor, Stl, which controls expression of most of the SaPI genes [14]. Following infection by a helper phage or induction of a helper prophage, SaPI de-repression is effected by specific, non-essential “moonlighting” phage proteins that bind to Stl, disrupting the Stl-DNA complex and thereby initiating the excision-replication-packaging (ERP) cycle of the island [15,16]. Different SaPIs encode different Stl proteins, so each SaPI commands a specific phage protein for its induction [15,16]. Since SaPIs require phage proteins to be packaged [17,18], this strategy couples the SaPI and phage cycles, but imposes a very significant transmission cost on the helper phages.

In previous work, we observed that different helper phages encoded allelic variants of the inducing genes with different affinity for the SaPI-encoded repressors [15]. Moreover, we also observed that phage mutants capable of forming plaques on SaPI-positive strains had mutations in the phage-coded inducing genes [15]. Here, we experimentally show that phages that fail to induce SaPIs as a result of spontaneous mutations of the inducing proteins are strongly favoured by selection, but that these mutants carry fitness cost in the absence of SaPIs. Propagation of SaPIs on these non-inducing phages results in strong selection of spontaneous SaPI *stl*-mutants that can be packaged and transferred by the evolved non-inducing phages, imposing a large transmission cost on the helper phages. Furthermore, bioinformatics data supports the view that SaPIs are an important selective pressure driving the diversity of both genes and gene content in *S*. *aureus* phages. Finally, to show the generality of this result we report similar experimental and bioinformatic results for *Enterococcus faecalis* phages. Taken together, our results suggest that helper and satellite viruses undergo extensive antagonistic coevolution.

## Results

### The SaPI inducers are under purifying selection in natural populations

To determine if SaPIs play an obvious role in phage evolution, we initially analysed the phage sequences deposited in GenBank and identified allelic variants of the phage-coded SaPIbov1, SaPI1 and SaPIbov2 inducing proteins, corresponding to the dUTPase (Dut), Sri and 80α ORF15-like proteins, respectively [15]. Representative examples of the different SaPI inducers are shown in S1 Fig. We tested the different selective forces that may have been shaping these proteins during their evolution *in vivo* by calculating and comparing the *d*_*N*_ *−d*_*S*_ values of the representative SaPI inducer genes (S1 Table). The *d*_*N*_ *−d*_*S*_, which measures the difference in substitutions rates between non-synonymous site (*d*_*N*_) and synonymous site (*d*_*S*_), is classically used as an indicator of selective pressure acting on a protein-coding gene. As is summarised in Table 1 and shown in S1 Table, all the *d*_*N*_ *−d*_*S*_ comparisons were significantly lower than 0 (*p* < 0.005), indicating that the SaPI inducers are under purifying selection.

**Table 1:** The phage-coded SaPI inducer proteins are under purifying selection^a^.

Pathogenicity island inducer	Overall mean *d*_*N*_ *−d*_*S*_ ^b^	z^c^	*P*-value^d^
***Staphylococcus aureus***			
**Dut (SaPIbov1)**	-0.139 ± 0.031	-4.532	< 0.0001
**Sri (SaPI1)**	-0.415 ± 0.127	-3.268	0.0005
**SaPIbov2**	-0.385 ± 0.148	-2.594	0.0047
***Enterococcus faecalis***			
**EfsCIV583**	-0.376 ± 0.211	-1.780	0.0357

^a^Table shows the statistical analysis of the *d*_*N*_ *−d*_*S*_ values obtained comparing the SaPI inducing proteins analysed in S1 Table.

^b^Represents the overall mean distance of the values shown in S1 Table (± standard error).

^c^ *z* = mean distance / standard error.

^d^Statistical significance of the null hypothesis H_0_: *d*_*N*_ *−d*_*S*_ = 0.

It is assumed that the main consequence of the purifying selection is a reduction in the level of variation present in the locus under selection, produced by the removal from the population of less-adapted variants. However, the existence of multiple alleles in the phage-coded SaPI inducers suggests the existence of an evolutionary force operating in opposite direction that maintains the diversity observed in the SaPI inducer proteins. In previous studies, we demonstrated that variants of the SaPIbov1 and SaPIbov2 derepressing proteins differentially induce the SaPIbov1 and SaPIbov2 cycles, respectively [15]. Moreover, we also demonstrated that the highly divergent region present in the Dut proteins (motif VI; S1 Fig) determines the capacity of the phages to induce the SaPIbov1 cycle by controlling the affinity between the SaPIbov1 Stl repressor and the Dut protein [15,16]. These results suggest that SaPIs could favour certain alleles in the phage population because they have reduced capacity to induce the SaPI cycles.

### Experimental evolution of SaPI resistance

To test the hypothesis that phages are under strong selection to resist SaPIs, we experimentally determined if the interaction with the SaPIs resulted in the evolution of phages carrying variants in the SaPI inducing proteins. Phage 80α was used as a model because it induces three different SaPIs: SaPIbov1, SaPIbov2 and SaPI1. Strains RN4220 (SaPI-negative; a control) or JP1996 (RN4220 derivative carrying SaPIbov1) were initially infected with phage 80α (1:1 ratio, see scheme in S2 Fig). The resulting lysates were then used to infect again the same strains and after the third passage phages were phenotypically characterised. The phage lysates obtained after the third passage in strain JP1996 (SaPIbov1-positive) were used to infect strain JP2129, an RN4220 derivative carrying SaPIbov2. After the third passage done in strain JP2129, the evolved phages were then used to infect JP2966, an RN4220 derivative carrying SaPI1. As a control, phages only infecting RN4220 were propagated and analysed through the experiment (see scheme in S2 Fig).

**Fig 1:**
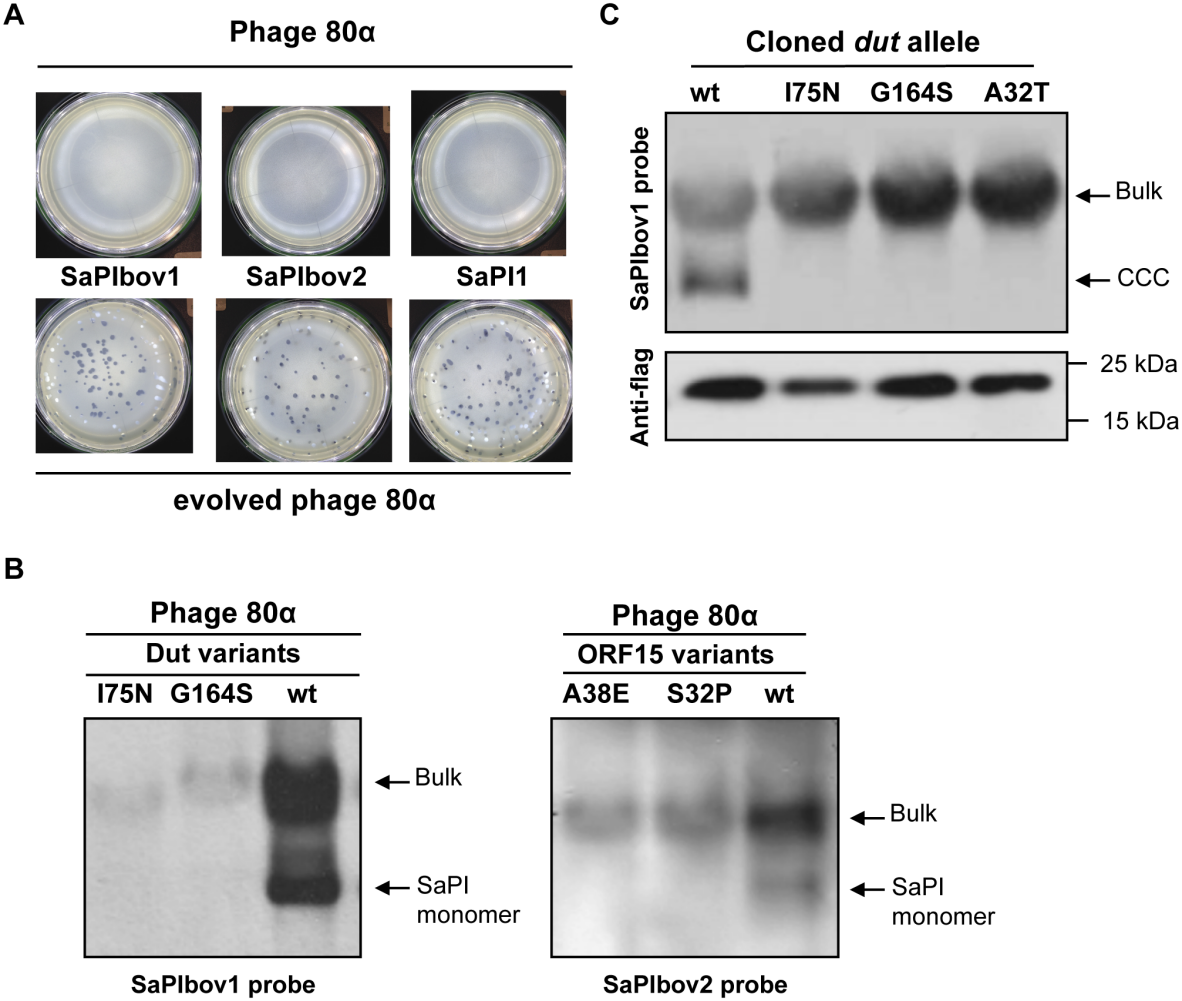
SaPI interference in evolved phages. (A) SaPI interference with phage reproduction. Approximately 10^8^ bacteria were infected with 100 p.f.u. of phage 80α (upper panel) or an evolved 80α derivative carrying mutation in all three SaPI inducers (lower panel), plated on phage bottom agar, and incubated 24 h at 32°C. (B) Induction of SaPIbov1 (left) or SaPIbov2 (right) by evolved 80α phages carrying mutations in the *dut* or ORF15 genes, respectively. Samples from the different lysogenic strains were isolated 60 min after induction with mitomycin C, separated on agarose and blotted with a SaPIbov1- or SaPIbov2-specific probe. Upper band is ‘bulk’ DNA, including chromosomal, phage and replicating SaPI; lower band is SaPI linear monomers released from phage heads. (C) SaPIbov1 excision and replication after induction of cloned *dut* genes from different evolved phages. A non-lysogenic derivative of strain RN4220 carrying SaPIbov1 was complemented with plasmids expressing 3xFlag-tagged Dut proteins. One millilitre of each culture (optical density (OD)_540nm_ = 0.3) was collected and used to prepare standard minilysates, which were resolved on a 0.7% agarose gel, Southern blotted and probed for SaPIbov1 DNA. In these experiments, because no helper phage is present, the excised SaPI DNA appears as covalently closed circular molecules (CCC) rather than the linear monomers that are seen following helper-phage-mediated induction and packaging. The upper panel is a Southern blot probed for SaPIbov1 DNA; the lower panel is a western blot probed with antibody (Sigma) to the Flag tag carried by the proteins.

We first determined growth of the ancestral and evolved phages. As observed in Fig 1A, while SaPIs blocked plaque formation by the ancestral 80α phage or by the phages evolved on the SaPI negative strain, they did not obviously interfere with the reproduction of the evolved phage mutant. These results demonstrate that phages evolved in the presence of SaPI no longer suffer reduced costs of SaPI parasitism. The most likely explanation of this reduction in cost is that the evolved phages were resistant to the SaPIs. This was investigated by generating lysogens from two evolved phages, which incidentally carried mutations in all three SaPI inducers (see below and S2 Table). Next we introduced into the different lysogens derivatives of SaPI1, SaPIbov1 or SaPIbov2 carrying a *tet*M marker, which facilitates transfer studies. The different SaPI-positive strains were then SOS (mitomycin C) induced and the capacity of the different phages to induce the SaPIs cycle was analysed. As shown in Fig 1B and S3 Table, none of the phage mutants induced the SaPIs. Moreover, uniquely the titre of the ancestral 80α phage, but not that from the evolved phages, was reduced by the presence of the islands (S3 Table). These experiments show that culturing phages with SaPIs results in the evolution of phage resistant to SaPIs (i.e no longer induce the SaPI cycle), and this resistance results in greatly increased phage proliferation on susceptible bacterial hosts.

### Genetic characterisation of resistance to SaPIs in experimental populations

From the aforementioned experiment, five 80α phages evolved after the third passage on strain JP1996 (SaPIbov1-positve) and 5 from the third passage on the RN4220 (SaPI-negative) branch were completely sequenced and analysed. Only phages that interacted with SaPIbov1 contained mutations in their genomes, which were in all cases located in the SaPIbov1 inducer gene *dut* (dUTPase). This result was further confirmed by sequencing the *dut* gene from other 120 evolved phages (60 infecting RN4220 and 60 infecting JP1996), obtained from 3 independent experiments. As summarised in Table 2 and shown in S2 Table, 100% of the phages infecting the SaPIbov1-positive strain showed mutations in the SaPIbov1-inducing gene *dut*. By contrast, no mutations were observed either in the other SaPI inducer genes, corresponding to *sri* and ORF15, or in the phages infecting the SaPIbov1-negative strain (Tables 2 and S2).

**Table 2:** Percentage of phages carrying mutations in the SaPI inducer genes after the serial passages against the SaPI-positive and SaPI-negative strains^a^.

No. phages analysed	Recipient strain	*dut*	Orf15	*sri*
		(SaPIbov1 inducer)	(SaPIbov2 inducer)	(SaPI1 inducer)
60	SaPIbov1-positive	100%	0%	0%
60	SaPIbov2-positive	100%	98.3%	0%
60	SaPI1-positive	95%	91.6%	100%
60	SaPI-negative	0%	0%	0%

^a^The table summarises the results from 3 independent experiments.

To determine the genetic basis of resistance to the other SaPIs, SaPIbov2 and SaPI1, the SaPI inducer genes from 120 evolved phages (from 3 independent experiments, 60 after interacting with SaPIbov2 and 60 after interacting with SaPI1) were analysed. The SaPI inducer genes were also sequenced from 60 phages that had only infected the SaPI-negative RN4220 strain (S2 Fig). As occurred with SaPIbov1, interaction with SaPIbov2 and SaPI1 selected for phages carrying missense and nonsense mutations in the SaPI inducer genes (Tables 2 and S2).

In previous work, we demonstrated that expression of the cloned SaPI inducing genes in a SaPI-containing strain was sufficient to induce the SaPI cycles [15]. As shown in Fig 1C, the cloned *dut* genes from the evolved phages did not induce SaPIbov1, while the wild-type gene did. As the Dut protein levels produced from these constructs are comparable (Fig 1C), this result confirms that the mutations present in the inducing genes are the cause of the inability of the evolved phages to de-repress the SaPI cycles.

### Costs of resistance in experimental populations

Given the high cost imposed by SaPIs on helper phages and the apparent ease at which they can evolve resistance, why isn’t resistance to SaPI exploitation ubiquitous? Part of the explanation might be that there are costs associated with resistance. Indeed, in the absence of the SaPIs both the phage titres and the phage plaque sizes were slightly but consistently reduced in the phage mutants, compared with the wt phage (S3 Table). Moreover, the number of phages carrying the wild-type versions of the SaPIbov1 and SaPIbov2 inducing genes increased in absence of the interference (Table 2). This putative cost was confirmed by competition experiments (in duplicate) among the ancestral 80α and two different evolved phages on the SaPI-negative host (RN4220). Two thousand p.f.u. of a mixed population (ratio 1:1) of the wt and one of the evolved phages was used to infect a plate containing 1 x 10^6^ RN4220 cells. Confluent phage plaques were collected, the lysate filtered and the procedure repeated four more times. After the fifth passage, 20 independent plaques from each of the different experiments were selected and the percentage of the phages under competition was evaluated by PCR and sequencing analyses of SaPI inducing genes. While the 80α wt and the evolved 80α phages were present in equal numbers in the mixed initial population, passages through the SaPI-negative RN4220 strain selected for the wt phage (*p* < 0.01; Table 3), confirming there is obvious cost to being resistant to single or multiple SaPIs in this experimental context.

**Table 3:** Fitness cost of the evolved phages.

Mutant phage^a^	Replicate	Percentage of wt phage^b^	
		Initial population	Final population
Dut I75N, ORF15 Q3*, Δ*sri*	1	12/20	19/20
	2	11/20	20/20
Dut G164S, ORF15 E40*, Sri C13Y	1	9/20	20/20
	2	10/20	19/20

^a^Mutant phages used to compete with the wt 80α phage.

^b^Shown is the ratio of the wt phages present in the initial or the final phage populations. In all cases, the presence of the wt phage were significantly increased at the end of the experiment (*p* < 0.01; Yates' chi-squared test).

### Experimental evolution of phage exploitation by SaPIs

We next determined whether SaPIs can in turn adapt to the presence of the experimentally evolved non-inducing phages. We made use of two different phage mutants that had evolved resistance to two SaPIs (SaPIbov1 and SaPIbov2) in the previous experiments (Table 4). The SaPIbov1 *tst*::*tet*M and SaPIbov2 *bap*::*tet*M islands, carrying a *tet*M marker that facilitates the SaPI transfer analyses, were introduced both in the two mutants and in the wt 80α phages. The different lysogenic SaPI positive strains were SOS (mitomycin C) induced and the islands transferred to the cognate recipient strains carrying the same phage that was present in the donor strain. After the transfer, the SaPI-positive strains were recollected and the procedure repeated 7 more times. After the eighth passage, the SaPI titre obtained was compared with that obtained with the original SaPIs. Remarkably, at the end of the experiment the SaPIs that interacted with the mutant phages increased their titres more than 10^4^-fold (Table 4), suggesting that the SaPIs had adapted to the presence of the SaPI insensitive phages. By contrast, the titres of those SaPIs interacting with the wt phage 80α did not change significantly through time. Note that these experiments were done four independent times and the obtained results were consistent in the parallel experiments.

**Table 4:** Transfer of the coevolved SaPI islands by the evolved phage mutants^a^.

Phage 80α	SaPI	Trial	SaPI titre^b^	
			Initial	Final
wt	SaPIbov1	1	1.23 x 10^8^	1.73 x 10^8^
wt	SaPIbov1	2	1.44 x 10^8^	1.04 x 10^8^
wt	SaPIbov1	3	2.07 x 10^8^	1.73 x 10^8^
wt	SaPIbov1	4	1.93 x 10^8^	1.04 x 10^8^
wt	SaPIbov2	1	1.21 x 10^8^	1.03 x 10^8^
wt	SaPIbov2	2	1.34 x 10^8^	1.40 x 10^8^
wt	SaPIbov2	3	1.33 x 10^8^	1.01 x 10^8^
wt	SaPIbov2	4	2.05 x 10^8^	9.47 x 10^7^
Dut S63I, ORF15 A38E, *sri* G 10983 A	SaPIbov1	1	1.21 x 10^3^ ^c^	9.13 x 10^7^
Dut S63I, ORF15 A38E, *sri* G 10983 A	SaPIbov1	2	1.03 x 10^3^ ^c^	1.44 x 10^8^
Dut S63I, ORF15 A38E, *sri* G 10983 A	SaPIbov1	3	9.29 x 10^2^ ^c^	9.66 x 10^7^
Dut S63I, ORF15 A38E, *sri* G 10983 A	SaPIbov1	4	1.643x 10^3^ ^c^	2.04 x 10^8^
Dut S63I, ORF15 A38E, *sri* G 10983 A	SaPIbov2	1	2.78 x 10^3^ ^c^	1.11 x 10^8^
Dut S63I, ORF15 A38E, *sri* G 10983 A	SaPIbov2	2	2.34 x 10^3^ ^c^	1.75 x 10^8^
Dut S63I, ORF15 A38E, *sri* G 10983 A	SaPIbov2	3	1.98 x 10^3^ ^c^	8.71 x 10^8^
Dut S63I, ORF15 A38E, *sri* G 10983 A	SaPIbov2	4	2.01 x 10^3^ ^c^	8.77 x 10^8^
Dut I75N, ORF15 Q3*, Δ*sri*	SaPIbov1	1	3.02 x 10^3^ ^c^	8.73 x 10^6^
Dut I75N, ORF15 Q3*, Δ*sri*	SaPIbov1	2	2.03 x 10^3^ ^c^	1.04 x 10^7^
Dut I75N, ORF15 Q3*, Δ*sri*	SaPIbov1	3	1.67 x 10^3^ ^c^	1.73 x 10^7^
Dut I75N, ORF15 Q3*, Δ*sri*	SaPIbov1	4	1.23 x 10^3^ ^c^	8.08 x 10^6^
Dut I75N, ORF15 Q3*, Δ*sri*	SaPIbov2	1	2.78 x 10^3^ ^c^	2.03 x 10^7^
Dut I75N, ORF15 Q3*, Δ*sri*	SaPIbov2	2	2.34 x 10^3^ ^c^	8.40 x 10^6^
Dut I75N, ORF15 Q3*, Δ*sri*	SaPIbov2	3	2.06 x 10^3^ ^c^	8.83 x 10^6^
Dut I75N, ORF15 Q3*, Δ*sri*	SaPIbov2	4	2.04 x 10^3^ ^c^	8.77 x 10^6^

^a^The means of results from three independent experiments are presented. Variation was within 5% in all cases.

^b^Transductants / ml of lysate, using RN4220 as recipient.

^c^This frequency is typical of transfer by generalized transduction and is not SaPI-specific.

To determine the genetic basis of SaPI adaption to “resistant” phage, 21 different colonies, randomly chosen from the different replicates, were individually analysed and the evolved SaPIs sequenced. As shown in S4 Table, the analysis of the individual colonies confirmed that the SaPIs had evolved in the presence of the mutant phages, but not in presence of the wt phage 80α. Importantly, the evolved SaPIs all had mutations in the *stl* gene. These mutations, located in the coding or in the promoter region of the *stl* gene (S4 Table), generated in all the cases an *stl*- mutant genotype. Thus, all the evolved SaPIs replicated autonomously in absence of any inducing phage. Previous work has shown that *stl* mutant SaPIs can be transferred by non-helper phages [14], and this is presumably why *stl* mutations massively increased SaPI transfer rates in the presence of the evolved “resistant” phages.

**Fig 2:**
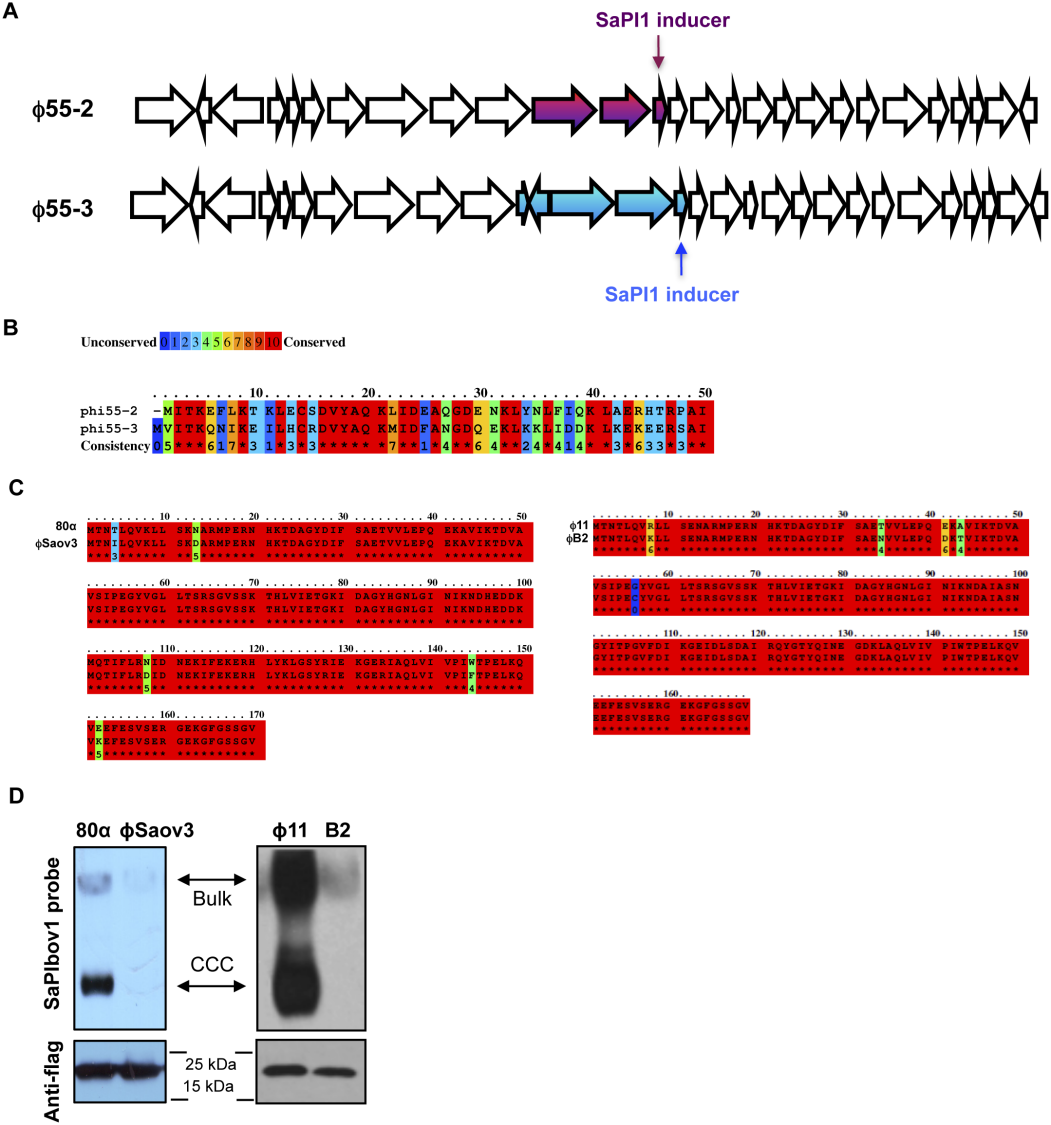
Coevolved SaPIs block phage reproduction. Plates carrying the SaPI-negative RN4220 strain, RN4220 derivatives carrying the original SaPIbov1 or SaPIbov2 islands, or RN4220 derivatives carrying evolved SaPIbov1 or SaPIbov2 islands were infected (<700 p.f.u. per plate) with two different evolved phage 80α. Genotype of the SaPIbov1 evolved island: A deleted from position 14119; genotype of the SaPIbov2 evolved island: deletion affecting residues from N122 to K168. Phage 1: 80α Dut I75N, ORF15 Q3*, Δ*sri*; Phage 2: Dut S63I, ORF15 A38E, *sri* G 10983 A.

Finally, we analysed if the coevolved SaPIs blocked reproduction of the evolved phages. As shown in Fig 2, this was the case. Thus, while the evolved phages were resistant to the presence of the original islands, the evolved SaPIs reduced phage reproduction.

### Evolved resistance to SaPIs in laboratory phage-typing stocks

Given the likely importance of SaPI-imposed selection, we speculated simple amplification of phages to obtain high titers of phage stock may have itself resulted in significant SaPI-imposed evolution. Phage collections have been traditionally used to type *S*. *aureus* strains. These collections are generated, maintained and amplified by infecting different propagating strains with specific phages. Interestingly, and as occurs in nature, most propagating strains carry uncharacterised prophages and SaPIs. In view of this, we hypothesised that the phage populations used for typing could contain mixed populations that have evolved in response to the SaPIs present in the propagating strains. To test this, we obtained 3 phage samples (ϕ29, ϕ52A and ϕ55) from a reference laboratory, and isolated, from each of these samples, five single phages, which were amplified using the non-lysogenic RN4220 strain. We used both the amplified phages obtained from the single plaques as well as the original phage populations to infect the non-lysogenic strain RN4220, as well as derivatives of this strain carrying SaPI1, SaPIbov1 or SaPIbov2. The rationale for this experiment was to compare the interference observed with these different phage samples. We hypothesised that if both samples had the same plating efficiency (interference) rate when infecting any of the SaPI-positive strains, both phage populations would be genetically homogenous (related to the SaPI inducers). By contrast, if a different behaviour was observed, and one of the samples infected the SaPI-positive strain better than the other sample, this would imply that the original population contained a mixed phage population that probably had evolved in response to the SaPI interference. Although the analysis of the ϕ29 and ϕ52A phage populations did not reveal any difference between the purified phages and those present in the samples from the reference laboratory, 1% of the original ϕ55 phage population generated plaques in the SaPI1-positive strain. By contrast, only 0.001% (1000 x reduction) of the purified ϕ55 phages generated plaques in this strain. This result suggested that the original phage lysate contained at least two different phage populations, evolved from a common ancestor, carrying variants of the SaPI1 inducing gene.

**Fig 3:**
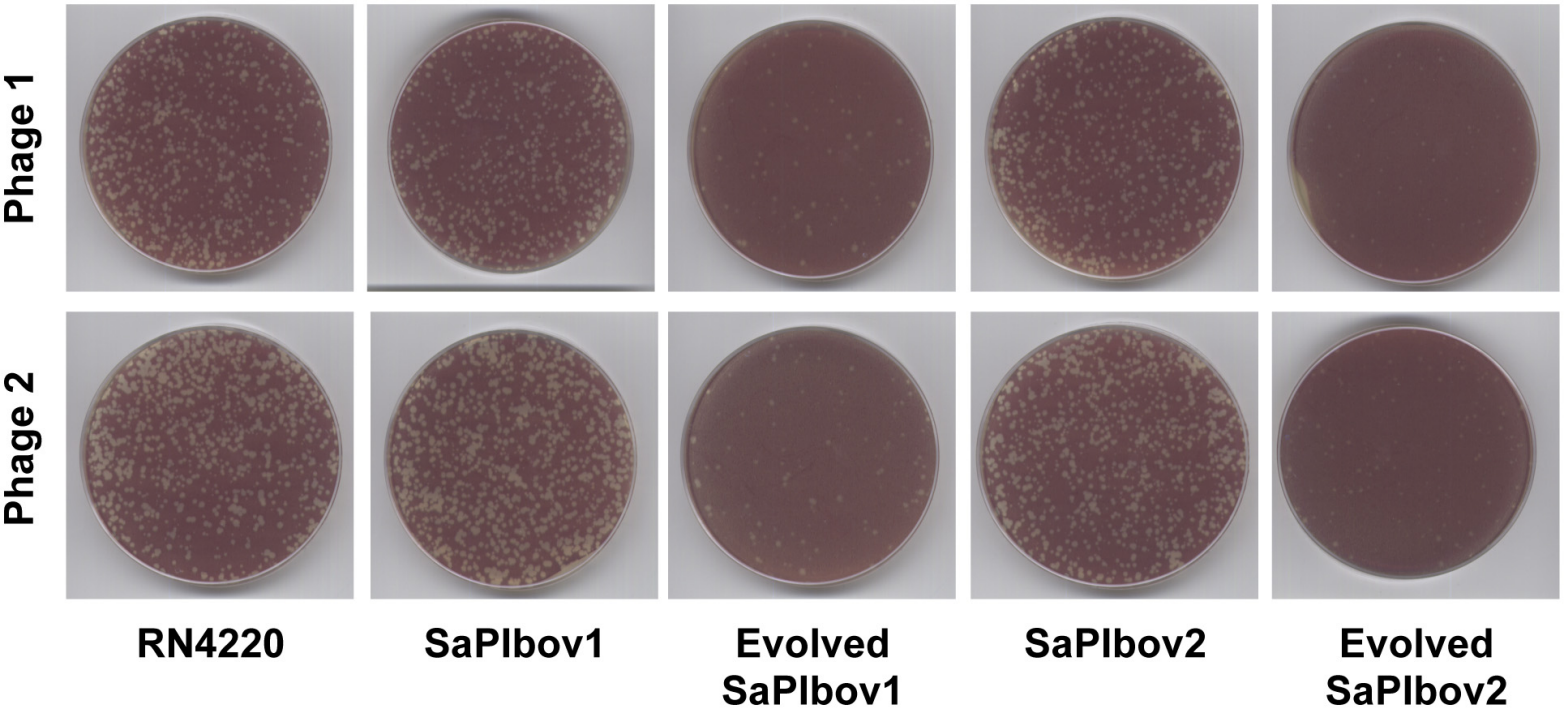
SaPI-driven phage evolution occurs *in vivo*. (A) Partial genetic maps of ϕ55–2 and ϕ55–3 (GenBank accession numbers KR709302 and KR709303, respectively). Arrows indicate predicted ORFs. Coloured arrows indicate the divergent region found in these phages, which include the SaPI1 inducing genes. (B) Lineup of Sri (SaPI1 inducer) protein sequences from phages ϕ55–2 and ϕ55–3, coloured according to relative sequence conservation at each position. Adapted from lineup generated by PRALINE [41]. The scoring scheme works from 0 for the least conserved alignment position, up to 10 (asterisk) for the most conserved alignment position. (C) Lineup of Dut (SaPIbov1 inducer) protein sequences from phages 80α and ϕSaov3 (left) or from phages ϕ11 and ϕB2 (right), coloured according to relative sequence conservation at each position. Adapted from lineup generated by PRALINE [41]. (D) SaPIbov1 excision and replication after induction of cloned *dut* genes from the natural mutant phages analysed in (C). A non-lysogenic derivative of strain RN4220 carrying SaPIbov1 was complemented with plasmids expressing 3xFlag-tagged Dut proteins, as indicated in Fig 1. The upper panel is a Southern blot probed for SaPIbov1 DNA; the lower panel is a western blot probed with antibody (Sigma) to the Flag tag carried by the proteins.

To test this, one of the previously purified phages infecting RN4220 but showing interference to the SaPI-positive strain was completely sequenced (ϕ55–2). One phage having no interference to SaPI1 was also purified and sequenced (ϕ55–3). The genome length of ϕ55–2 is 41,898 bp, containing the information for approximately 81 ORFs of 50 or more codons, and is deposited in GenBank under accession number KR709302. The genome length of ϕ55–3 is 42,309 bp, with approximately 83 ORFs, and is deposited in GenBank under accession number KR709303. Both ϕ55–2 and ϕ55–3 belong to a class of related staphylococcal *Siphoviridae* [19]. Overall, both phages are >99.9% identical except for a divergent region of ∼1800 bp that contains the gene coding for the SaPI1 inducer (Fig 3A). In the ϕ55–2 phage, this region encodes 3 ORFs, the last one being the SaPI1 inducer. By contrast, phage ϕ55–3 encodes 5 different ORFs, including a variant of the SaPI1 inducer (Fig 3B). Since the ORFs present in phage ϕ55–3 were also contained in other *S*. *aureus* phages, one of many plausible explanations for the differences between these two phages is that recombination occurred between ϕ55–2 and a prophage residing in the propagating strains. To verify that the aforementioned changes observed in phage ϕ55–3 were not generated during the purification and amplification of the phage, PCR experiments with specific oligonucleotides for each of the phages were performed, using DNA samples obtained from the original phage population (without amplification).

To confirm that the mutations present in phage ϕ55–3 conferred an advantage for the phage in the presence of a SaPI1-positive strain, competition experiments (in triplicate) were performed in which a SaPI1-negative or a SaPI1-positive strain were infected (phage:bacteria ratio 1:3) with a mixed population (1:1) of the ϕ55–2 and ϕ55–3 phages. The lysates obtained from each experiment were used to infect again the same strains, and after the third passage, the number of the ϕ55–2 and ϕ55–3 phages was evaluated by PCR using oligonucleotides that specifically recognise the different allelic variants of the phage coded SaPI1 inducers. While both phages were present in equal numbers after infecting the SaPI-negative strain (ϕ55–2: 55%; ϕ55–3: 45%), passages through the SaPI1-positve strain selected for ϕ55–3 (>95%).

### SaPI-driven phage evolution occurs in nature

The previous results suggested that it would be possible to find closely related phages encoding different alleles of the SaPI inducers as a consequence of the phage interaction with the SaPIs. To address this, we initially performed a phylogenetic analysis of 33 randomly selected staphylococcal phages (S3A Fig). Next, we compared the SaPI inducer sequences from closely related phages. As hypothesised, the genes coding for the SaPI inducers’ proteins represent a source of variation among closely related phages. S3B Fig shows representative examples of these comparisons. Moreover, since distantly related phages encode the same SaPI derepressing protein, our analysis revealed that SaPI inducer diversity is independent of phage phylogeny (S1A, S1C, S1E and S3 Figs).

Interestingly, this analysis also revealed one additional strategy by which phages might avoid SaPI repression, namely, losing the genes encoding for the SaPI inducers. Thus, the SaPI1 inducer *sri* was absent in phages ϕ11, ϕPVL or ϕNM3 and the SaPIbov2 inducer was not present in phages ϕ11, ϕNM2, ϕPVL-CN125, ϕPVL, ϕNM3 or ϕ52a, although closely related phages coded for the missing inducers (S3A Fig). With regards to the SaPIbov1 inducer (trimeric Dut), it is absent in phages ϕ69, ϕNM1, ϕNM2, ϕPVL108, and ϕ55. Surprisingly, instead of the trimeric form, these phages code for a dimeric Dut, which based on the structure of some homologue proteins deposited in the protein data bank (PDB), we predict to be functionally related but structurally completely different. Why some phages encode a dimeric or a trimeric Dut is under study. This analysis suggests SaPI-imposed selection can drive significant and rapid evolutionary change in natural phage populations.

Since our laboratory passage experiments suggested that only a few mutations are required to generate phages that escape from SaPI interference, we hypothesised that a similar process will have occurred in nature. To test this, we looked for proteins with high (but not complete) similarity to the 80α or ϕ11 Duts. Two promising candidate were the Dut proteins encoded by the prophages ϕSaov3 and B2 (accession numbers YP_005736587 and ERS400827), which have only 5 amino acid changes compared with the 80α and ϕ11 Duts, respectively (Fig 3C). As shown in Fig 3D, the ϕSaov3 and B2 Dut variants were unable to induce the SaPIbov1 cycle, validating the results obtained with the *in vitro* evolved phages.

These variants, however, are not widespread in nature. Since the SaPI inducers are moonlighting proteins with a relevant role in the phage biology [20], we hypothesised that these variants have probably also affected their function for the phage. This was analysed by testing the enzymatic activity of 3 Dut variants (the natural Dut B2 and the evolved Dut 80α I75N and Dut 80α G164S). This analysis revealed that the B2 Dut protein is insoluble and completely inactive, while the two evolved variants had significantly reduced (*p* < 0.01, Student’s *t*-test) their dUTPase activity (S4 Fig). In addition, of note is the existence in the evolved phages of some *dut* genes carrying nonsense and frameshifts mutations, which encode non-functional proteins (S2 Table). This loss of function probably explains why these variants do not exist or are not widespread in nature.

### The EfCIV583 island drives phage evolution in *Enterococcus faecalis*

To demonstrate that phage-inducible chromosomal islands are important for phage evolution and ecology in general, we analysed the interaction between the enterococcal pathogenicity island EfCIV583 and its inducing phage ϕ1 [21]. To do this, we initially demonstrated that the EfCIV583 element interferes with the phage ϕ1 reproduction (S5 Fig). Next, strains JP10983 or JP10982 (JP10983 derivative carrying EfCIV583) were infected with phage ϕ1, as previously reported in the analysis of the SaPI-phage interaction. After the third passage, 6 phages (3 infecting JP10983 and 3 infecting JP10982) were completely sequenced and analysed. Only those phages that had interacted with EfCIV583 contained mutations in their genomes, always located in the ϕ1 *xis* (EF0309) gene (S5 Table). Remarkably, and in a parallel study, we have demonstrated that the ϕ1 *xis* gene is the inducer for the EfCIV583 island [22].

**Fig 4:**
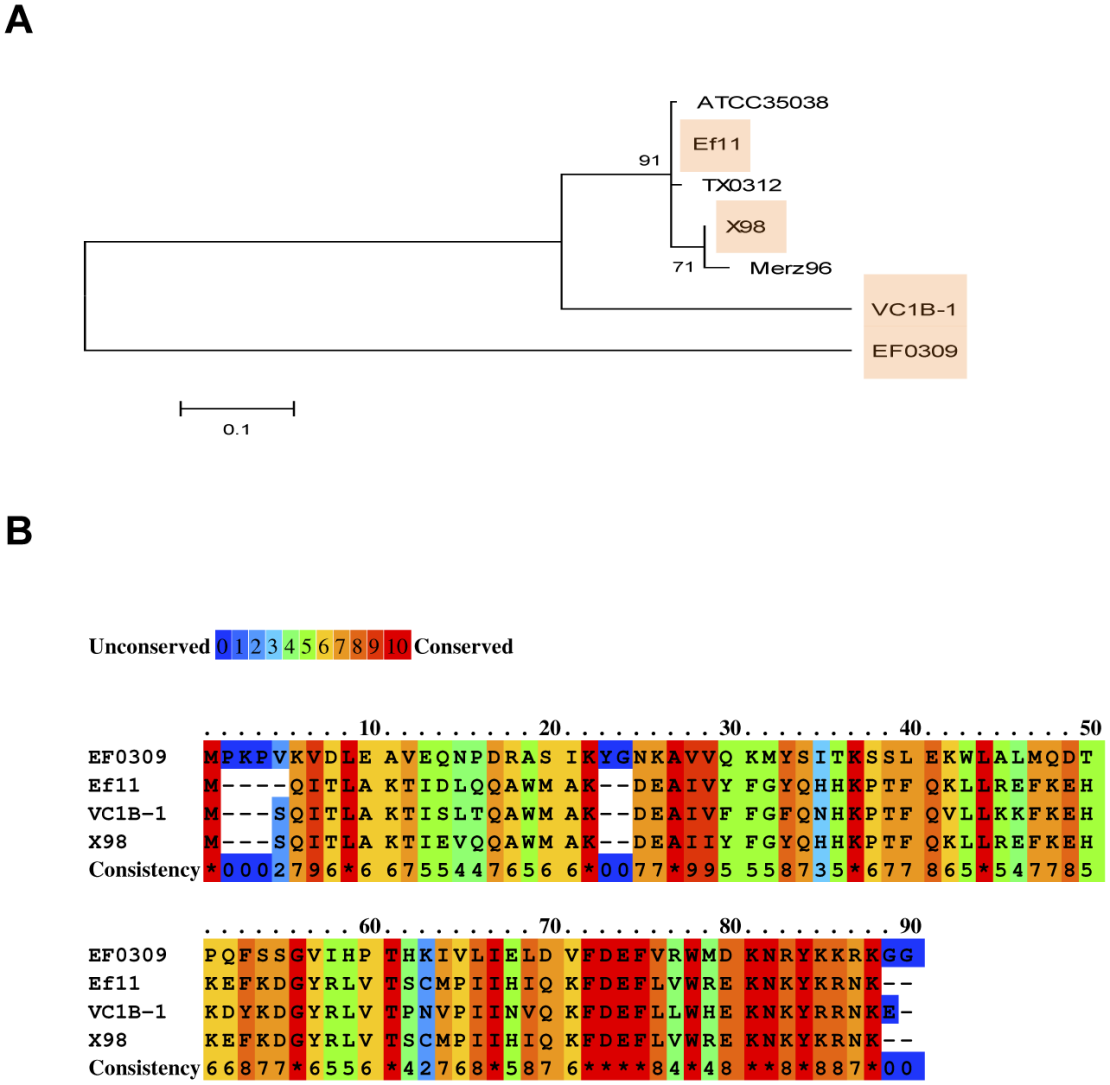
The enterococcal EfCIV583 island drives phage evolution. (A) Nearest neighbour tree of EfsCI_V583_ inducer proteins generated by MEGA5 [36]. Numbers indicate the bootstrap value. Shaded are the proteins characterised in this study. (B) Lineup of selected EfCI_V583_ inducer protein sequences from different enterococcal phages and prophages, coloured according to relative sequence conservation at each position. Adapted from lineup generated by PRALINE [41]. Accession numbers EfsCIV583 inducer proteins: EF0309 (AAO80172); Ef11 (YP_003358829); X98 (WP_002381619); VC1B-1 (EPI33180).

Next, to test if this process is relevant *in vivo*, we analysed whether related enterococcal phages encoded allelic variants of the EfCIV583 inducer, and if these variants are under purifying selection. As shown in Fig 4 and Tables 1 and S1, this was the case, confirming that satellite phages are a major force driving phage evolution.

## Discussion

The significance of coevolution between prokaryotes and their viruses [23,24], and between viruses and their associated viral defective interfering particles [29] is well established. Here, we investigate coevolution between helper and satellite viruses of *S*. *aureus*. We confirm previous results that parasitism by SaPIs, which exploit phages for their own transmission, impose a massive growth rate cost on the phages [23–25]. We then show real-time evolution of phage resistance against SaPIs, and in turn the evolution of SaPI exploitation of “resistant” phages. We identify the genetic basis of experimentally evolved resistance and exploitation, and confirm the importance of SaPI-imposed selection on phage evolution in both natural populations of *S*. *aureus* phages through bioinformatic analyses and in laboratory populations of *S*. *aureus* phages used in phage typing. Finally, we report comparable experimental and bioinformatics results in *E*. *faecalis*.

We have previously demonstrated that one of the key features of the SaPIs is to interfere with helper phage reproduction, using a variety of mechanisms. These include *i*) blocking of phage DNA packaging by expression of the SaPI-coded Ppi protein, which interferes with the phage coded TerS protein [5]; *ii*) diversion of phage proteins to produce the SaPI-specific particles [5]; *iii*) expression of the PtiA homologs [6], which block phage growth by binding to the helper phage Ltr proteins [26,27], directly inhibiting their ability to activate phage late gene transcription; or *iv*) carriage by the SaPIs of the phage *cos* or *pac* sites in order to compete with the inducing phages to be packaged in the phage particles [28,29]. In this work we describe two complementary strategies by which the phages evolve to overcome the SaPI interference: one involves the generation of allelic variants in the SaPI de-repressing proteins with lower affinity for SaPI coded Stl repressor; the other, even more drastic, involves complete loss of the phage-encoded SaPI inducing genes. Since SaPI interference depends on the induction of the SaPI cycle, both strategies select for non-inducing phages that are not affected by the presence of a quiescent SaPI integrated in the bacterial chromosome. Crucially, we show that SaPIs in turn adapt to resistant helper phage by loss of function of the global Stl repressor that removes the need for specific phage proteins for induction, allowing transmission of SaPIs by any infecting phages rather than specific helper phages [14].

While our results show that coevolution between satellite and helper viruses can occur very rapidly, it opens up a number of key questions. Specifically, how is the intimate association between phages and SaPIs maintained given that SaPI adaptation resulted in the loss of the need of specific helper phages for induction? First, there are massive costs associated with loss of function of the SaPI global repressor. As previously reported, mutations in the *stl* gene severely affects bacterial physiology and growth, probably because of the uncontrolled replication of the *stl* mutant SaPIs [14]. This explains why the SaPIs characterised to date encode a functional Stl protein that block the SaPI cycle in the absence of the helper phage, although it is entirely feasible that de-repressed SaPIs can be favoured by selection in natural populations, at least for short periods of time. Second, there are costs associated with loss or alteration of the phage inducing proteins, as apparent from the increase in the number of phages carrying the wild-type versions of the SaPIbov1 and SaPIbov2 inducing genes in the absence of the interference caused by these islands, as well as the results from competition experiments between wildtype and mutant phage. As a result of such costs, wild-type phages are likely to be maintained in the population, further weakening selection for SaPI *stl* mutants. These costs of SaPI resistance presumably arise because the phage-coded SaPI inducers are proteins that perform their functions through protein-protein interactions with other phage- or bacterial-coded proteins [30], and that the Stl repressors have merged the structure of the partners to which the SaPI inducers interact in order to be targeted [16]. Although this has not been demonstrated yet for the SaPIbov1 (*dut*) and SaPIbov2 (80α ORF15) inducers, the Sri protein (SaPI1 inducer) interact with the cellular DnaI protein inhibiting staphylococcal replication [20].

Based on this and previous in vitro results discussed above, one type of dynamic of continual phage-SaPI coevolution in nature may therefore be cycles of the following specific events: i) Phages evolve SaPI resistance by alteration or loss of the inducing protein, assuming that the short term benefits of SaPI resistance outweigh fitness costs (if any) associated with changes in the inducing protein; ii) SaPIs respond by loss of the need to be induced by a helper phage, again assuming benefits to the SaPI of being transmitted to new hosts outweigh the costs of reducing the fitness of hosts they infect; iii) As a result of the mutant SaPIs being able to exploit both the original and mutant “resistant” phages, phages with the original unaltered SaPI inducing proteins are able to outcompete mutant phages because of the costs associated with altered inducing proteins which no longer confer resistance; and iv) SaPIs that require induction by helper phage proteins can now outcompete SaPIs that do not require an inducer because there are large numbers of inducing phage present, starting the cycle again. This type of coevolutionary dynamic can be described as range fluctuating selection [31], and can arise when increased resistance and infectivity ranges are associated with increased fitness costs [31]. Note that ranges here refer to the number of SaPI and phage genotypes that can be resisted and infected, respectively.

The high diversity of phage inducing protein alleles suggests however alternative coevolutionary dynamics are operating in nature in addition to or instead of the model described above. Specifically, SaPI-imposed selection may cause diversifying selection if SaPIs adapt to changes in inducing proteins by switching to exploit the modified or an alternative protein, rather than losing the need for specific helper phages, and hence evolving more general infectivity. Consistent with this model, SaPIs, encoding different Stl repressors, have acquired the ability to exploit entirely unrelated phage proteins as antirepressors, and SaPIs tend to be induced by single rather than multiple proteins [15]. This coevolutionary dynamic can be described as specialism fluctuating selection, and again can arise when mutations that confer host or parasite generalism are too costly [32].

We have also demonstrated that another member of the PICI family of mobile genetic elements, the EfCIV583 island, drives phage evolution. Since one of the key features of the PICI elements is to interfere with the phage biology, we anticipate that these elements will have developed multiple interference functions that will have to be overcome by the helper phages in order to prevent PICI interference. Since PICI elements have been found in most Gram-positive bacteria, we anticipate that the strategies reported here by which the phages evolve in response to the SaPIs are widespread in nature.

Our work shows that virus satellites associated with both *S*. *aureus* and *E*. *faecalis* can have important ecological and evolutionary implications on their helper viruses. Crucially, these findings add another layer of complexity to the increasingly recognised role of coevolution between viruses and their bacterial hosts in driving ecological and evolutionary dynamics of these organisms [33]. For example, whether or not phages are resistant to the dominant satellite viruses they encounter at a given point in time, will in turn determine the how phages affect their bacterial hosts. Arguably, it may be necessary to explicitly consider the role of satellite viruses to understand the structure of any natural microbial community, over and above their well-recognised role in horizontal gene transfer.

## Materials and Methods

### Bacterial strains and growth conditions

The bacterial strains used in these studies are listed in S6 Table. The procedures for preparation and analysis of phage lysates, in addition to transduction and transformation of *S*. *aureus*, were performed essentially as previously described [14,28,34]. *E*. *faecalis* lysates were obtained and prepared as previously indicated [21].

### DNA methods

General DNA manipulations were performed using standard procedures. The plasmids and oligonucleotides used in this study are listed in S7 and S8 Tables, respectively. The labelling of the probes and DNA hybridization were performed according to the protocol supplied with the PCR-DIG DNA-labelling and Chemiluminescent Detection Kit (Roche). Southern and western blots experiments were performed by standard procedures [15].

### Experimental evolution of SaPI resistance

To test if the phages evolved in the presence of the different islands, 5 x 10^7^ cells of the SaPIbov1-positive strain JP1996 were initially infected with phage 80α (1:1 ratio). Once the culture lysed, which occurred normally 4–5 hours post-infection, the resulting phage population was titred and used to infect again the SaPIbov1-positive strain, using always the aforementioned cells:phage ratio. The phage lysates obtained after the third passage done in strain JP1996 were used to infect strain JP2129, an RN4220 derivative carrying SaPIbov2. After the third passage done in this strain (JP2129), the evolved phages were then used to infect JP2966, an RN4220 derivative carrying SaPI1. As a control, phages only infecting RN4220 were propagated and analysed through the experiment (see scheme in S2 Fig).

### Expression of the SaPI inducing proteins

The different phage genes under study were PCR amplified using oligonucleotides listed in S8 Table. PCR products were cloned into pCN51 [35] or pET28a (*E*. *coli*; Novagen), and the resulting plasmids (S7 Table) were sequenced and introduced into the appropriate recipient strains (S6 Table).

### Analysis of selective pressures

Selective pressures operating on SaPI or EfsCIV583 inducers were analysed using the average difference between substitution rates per nonsynonymous and synonymous sites, *d*_*N*_−*d*_*S*_, over all pairs of sequences. A zero value indicates neutral evolution (no selection); greater than zero supports positive directional selection, and lower than zero is taken as evidence of purifying (stabilising) selection. Substitution rates were computed using Nei-Gojobori modified method with Jukes-Cantor correction (with transitions to transversions bias equal to 0.66). Standard errors were estimated by a bootstrap procedure (1000 replicates). These analyses were done using MEGA5 [36].

Sequence alignments were screened for the presence of recombination using all the algorithms implemented in the RDP4 program [37] as well as the SBP and GARD [38] algorithms implemented in the Datamonkey server (www.datamonkey.org). No evidence for recombination was found for the SaPI1 (Sri), SaPIbov2 and EfsCI_V583_ inducers. However, a weak evidence was found by RDP4 for the SaPIbov1 inducers (Duts), but this was not supported by the other algorithms. Thus, we decided not to consider any Dut sequence as a truly recombinant.

### Reconstruction of phage phylogeny

The genome of selected phages was aligned using MAUVE version 2.3.1 [39] with its default parameters. Genetic relatedness among phages in the presence of possible recombination events and of parallel evolutionary changes was evaluated through a NeighborNet network reconstructed with SplitsTree version 4.11.3 [40]. The Jukes-Cantor model of nucleotide substitutions was used to estimate genetic divergences among pairs of sequences. Statistical support for the edges in the split graph was evaluated with 1000 bootstrap resamplings of the sequence data.

### Enzyme assays

dUTPase activity was assayed using His(6)-dUTPase proteins purified after expression in *E*. *coli*, using standard procedures. Enzyme assays were performed using the EnzCheck Pyrophosphate Assay Kit (Molecular Probes), as previously reported [16].

## Supporting Information

S1 Fig Sequence comparison of the phage coded SaPI inducers. (A, C and E) Phylogenetic network of the SaPI inducer coding genes generated by SplitsTree version 4.11.3 (Huson et al. 2006). Numbers in the branches indicate the bootstrap support for each bipartition. Branch lengths are proportional to genetic differences among phage-coded SaPI inducers. Shaded are the proteins characterised in this study. (B, D and F) Lineup of selected SaPI inducer protein sequences from different *S*. *aureus* phages and prophages, coloured according to relative sequence conservation at each position. Adapted from lineup generated by PRALINE. Accession numbers Dut proteins: 80 (YP_001285346); Rosa (YP_240373); 71 (YP_240446); ETA2 (YP_001004294); PVL108 (YP_918921); NM3 (YP_908820); 85 (YP_239795); 3A (YP_239989); PVL-CN125 (YP_002939700). SaPIbov2 inducers: ROSA (YP_240350); 85 (YP_239776); 80 (YP_001285329); Strain DAR390 (EXY00034); Strain PLAC6004 (EVF70071). Sri proteins: Strain A6300 (EEV76885); Strain Mu50 (BAB57032); 85 (YP_239784); Strain 21252 (EHO92899); Strain M79256 (EXP87181); 80 (YP_001285336); 55 (YP_240514).
(PDF)

S2 Fig Schematic representation of the experiment performed to analyse the role of the SaPIs driving phage evolution. (PDF)

S3 Fig Staphylococcal phage sequences comparison. (A) The genetic relatedness among the *S*. *aureus* phages in the presence of possible recombination events and of parallel evolutionary changes is shown. Shaded are the proteins characterised in this study. Accession numbers phages: 80 (DQ517338); 53 (AY954952); 11 (AF424781); 85 (AY954953); 69 (AY954951); NM1 (DQ530359); NM2 (DQ530360); ETA2 (AP008953); 12 (AF424782); PVL (AB009866); PVL108 (AB243556); 42E (AY954955); 52A (AY954965); ROSA (AY954961); PVL-CN125 (FJ713816); 77 (AY508486); 47 (AY954957); P954 (GQ398772); NM3 (DQ530361); MR11 (AB370268); 187 (AY954950); SLT (AB045978); tp310-3 (EF462199); 29 (AY954964); 71 (AY954962); 2638A (AY954954); EW (AY954959); 37 (AY954958); PH15 (DQ834250); 13 (AF424783); 55 (AY954963); 80 (DQ908929); MR25 (AB370205). (B). Lineup of the SaPI inducer proteins selected in (A). Adapted from lineup generated by PRALINE. Accession numbers Dut proteins: SLT (NP_075491); 187 (YP_239558); 12 (NP_803328); 2638A (YP_239842); 29 (YP_240597); ROSA (YP_240373); PVL-CN125 (YP_002939700); tp310-3 (YP_001429990). SaPIbov2 inducers: EW (YP_240202); PH15 (YP_950707); 37 (YP_240124); 29 (YP_240582.1); ROSA (YP_240350); MR25 (YP_001949812); ETA2 (YP_001004275). Sri proteins: 52A (YP_240656); 55 (YP_240514); tp310-3 (ERS400827); PVL-CN125 (YP_002939693.1).
(PDF)

S4 Fig Enzymatic activity for Dut80α and mutants. The graph shows the Vmax, expressed in nmoles of dUTP hydrolysed in one second for 1μg of protein, measured for Dut80α and mutants. In all cases, the experiments were done in triplicate. Student’s *t*-test was used to compute *p* values for group comparisons; differences that are statistically significant are indicated by an asterisk (*p* < 0.01). All error bars show s.e.m.
(PDF)

S5 Fig EfCIV583 interference with phage reproduction. Approximately 10^8^ bacteria were infected with 400 p.f.u. of phage 1, plated on phage bottom agar, and incubated 24h at 32°C. Plates were stained with 0.1% TTC in TSB and photographed.
(PDF)

S1 Table Estimates of codon-based evolutionary divergence between the phage coded SaPI inducer sequences. (PDF)

S2 Table Mutations present in the phage-coded SaPI inducing genes. (PDF)

S3 Table Effect of phage mutations on phage and SaPI titres. (PDF)

S4 Table Mutations present in the coevolved SaPIs. (PDF)

S5 Table Mutations present in the phage ϕ1 Xis protein. (PDF)

S6 Table Strains used in this study. (PDF)

S7 Table Plasmids used in this study. (PDF)

S8 Table Primers used in this study. (PDF)
