# Supplementary figures and images for: Virus Satellites Drive Viral Evolution and Ecology
Source: PLoS Genet. 2015 Oct 23;11(10):e1005609. doi: 10.1371/journal.pgen.1005609 (PMC4619825; doi:10.1371/journal.pgen.1005609)

Dut (SaPIbov1 inducer)

A

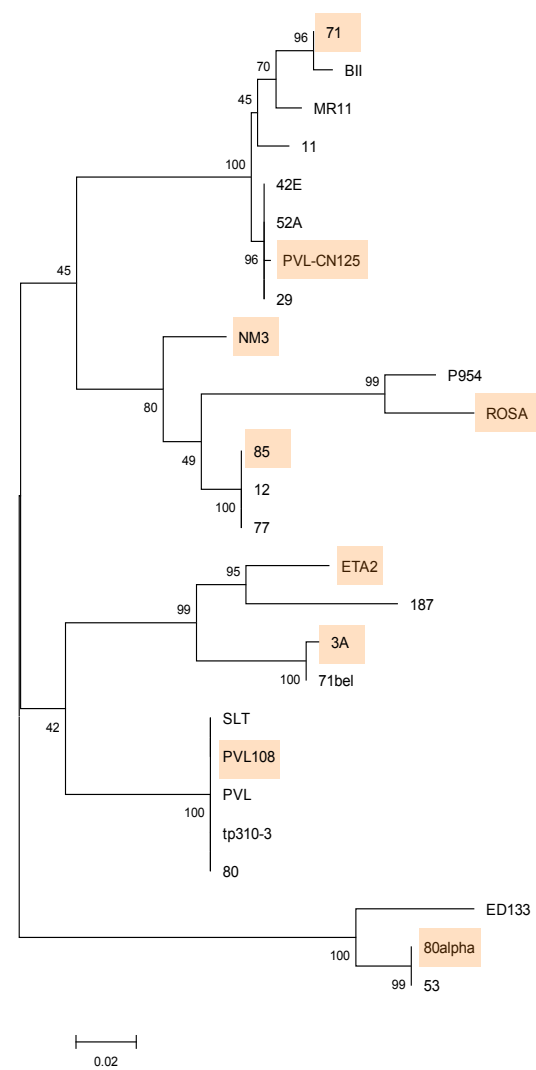

B

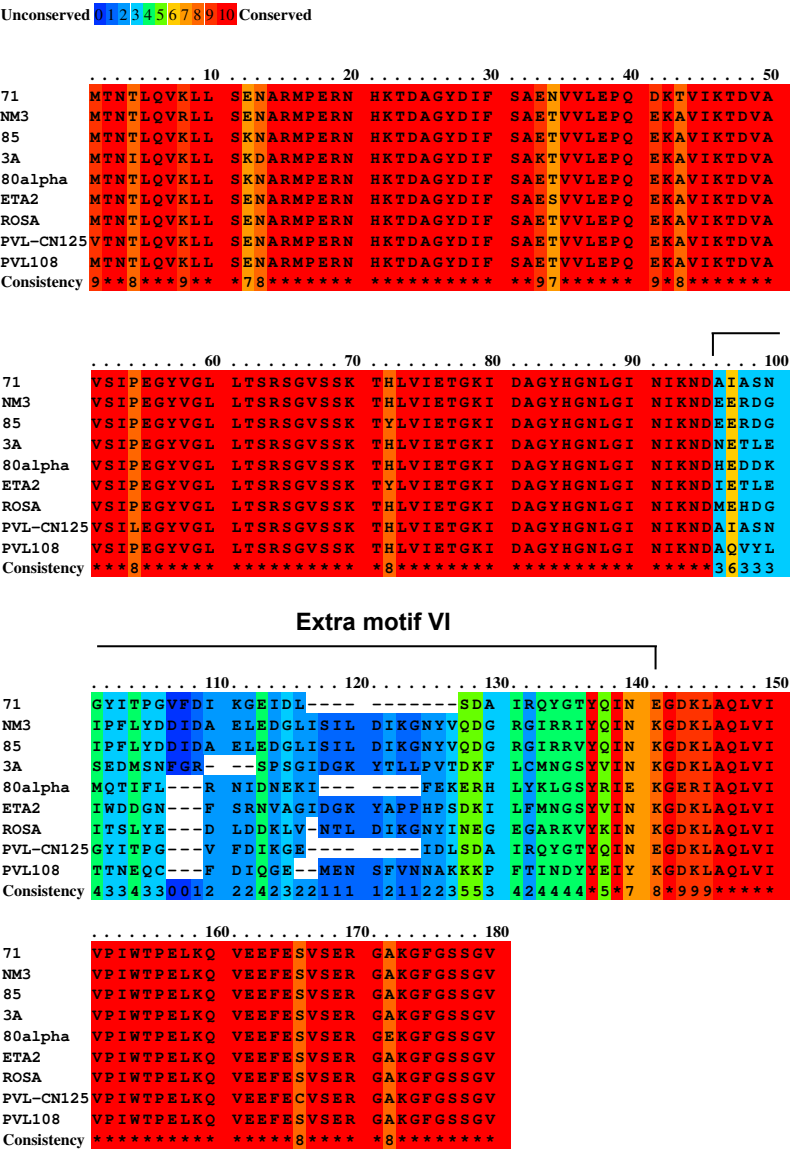

Sri (SaPI1 inducer)

C

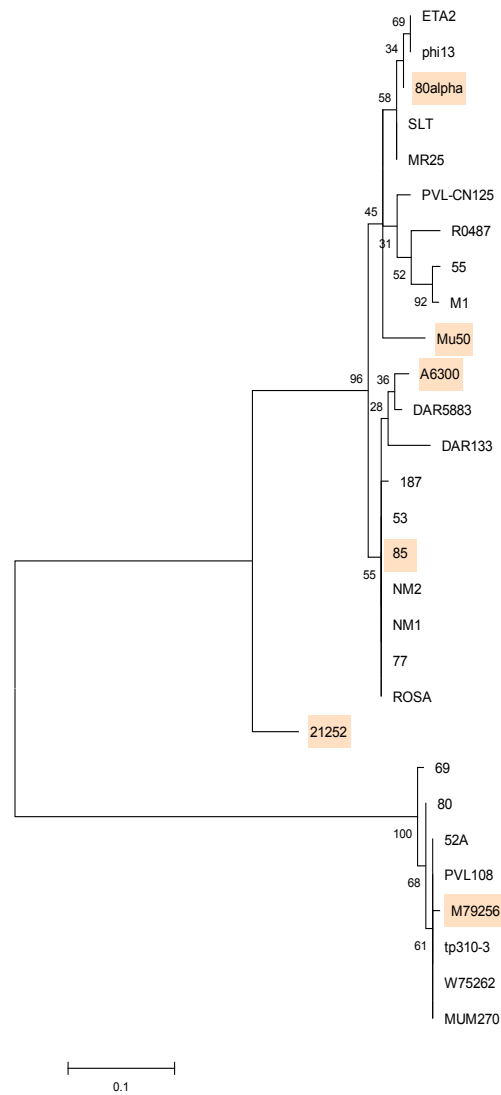

D

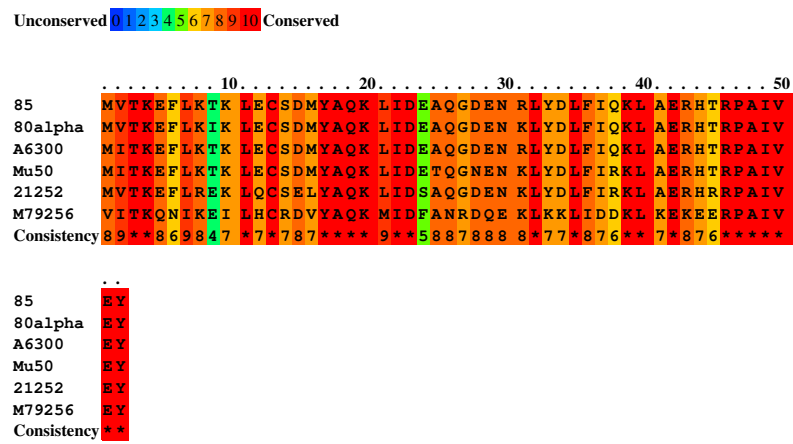

## E

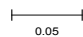**F**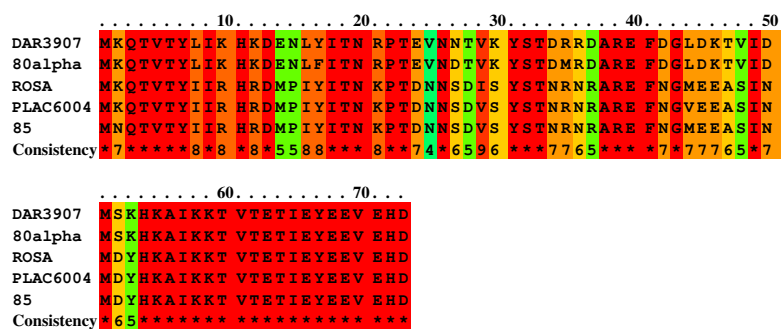

Supplement: S1 Fig — (A, C and E) Phylogenetic network of the SaPI inducer coding genes generated by SplitsTree version 4.11.3 (Huson et al. 2006). Numbers in the branches indicate the bootstrap support for each bipartition. Branch lengths are proportional to genetic differences among phage-coded SaPI inducers. Shaded are the proteins characterised in this study. (B, D and F) Lineup of selected SaPI inducer protein sequences from different S. aureus phages and prophages, coloured according to relative sequence conservation at each position. Adapted from lineup generated by PRALINE. Accession numbers Dut proteins: 80 (YP_001285346); Rosa (YP_240373); 71 (YP_240446); ETA2 (YP_001004294); PVL108 (YP_918921); NM3 (YP_908820); 85 (YP_239795); 3A (YP_239989); PVL-CN125 (YP_002939700). SaPIbov2 inducers: ROSA (YP_240350); 85 (YP_239776); 80 (YP_001285329); Strain DAR390 (EXY00034); Strain PLAC6004 (EVF70071). Sri proteins: Strain A6300 (EEV76885); Strain Mu50 (BAB57032); 85 (YP_239784); Strain 21252 (EHO92899); Strain M79256 (EXP87181); 80 (YP_001285336); 55 (YP_240514). (PDF) [file pgen.1005609.s001.pdf]

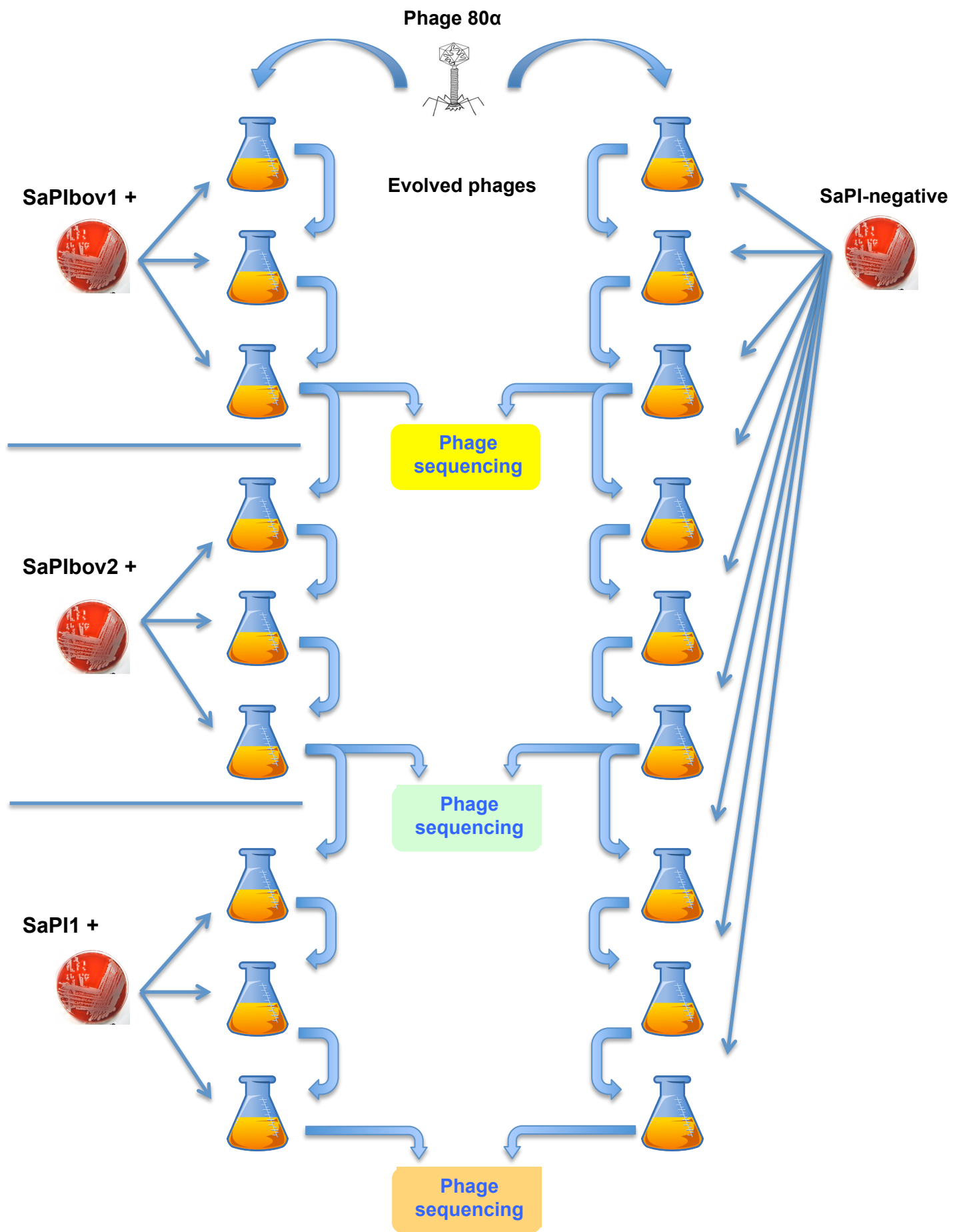

Supplement: S2 Fig — (PDF) [file pgen.1005609.s002.pdf]

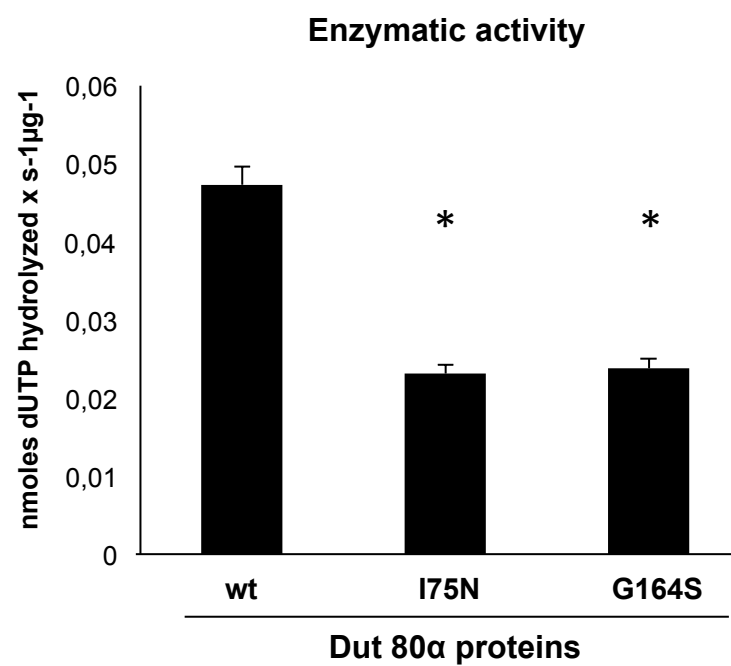

Supplement: S4 Fig — The graph shows the Vmax, expressed in nmoles of dUTP hydrolysed in one second for 1μg of protein, measured for Dut80α and mutants. In all cases, the experiments were done in triplicate. Student’s t-test was used to compute p values for group comparisons; differences that are statistically significant are indicated by an asterisk (p < 0.01). All error bars show s.e.m. (PDF) [file pgen.1005609.s004.pdf]

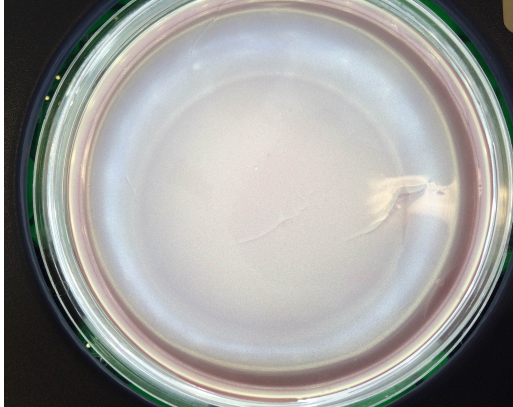

**EfsCIV583-positive**

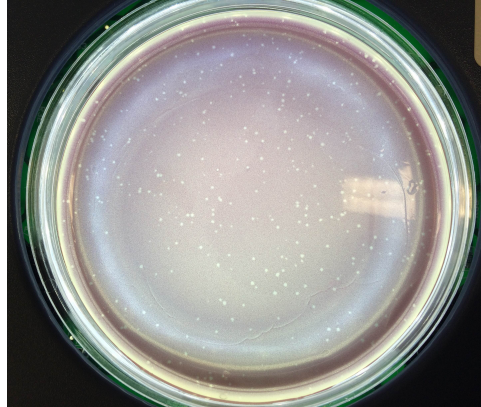

**EfsCIV583-negative**

Supplement: S5 Fig — Approximately 108 bacteria were infected with 400 p.f.u. of phage 1, plated on phage bottom agar, and incubated 24h at 32°C. Plates were stained with 0.1% TTC in TSB and photographed. (PDF) [file pgen.1005609.s005.pdf]
