# Supplementary material for: Virus Satellites Drive Viral Evolution and Ecology
Source: PLoS Genet. 2015 Oct 23;11(10):e1005609. doi: 10.1371/journal.pgen.1005609 (PMC4619825; doi:10.1371/journal.pgen.1005609)
Supplement: S3 Fig — (A) The genetic relatedness among the S. aureus phages in the presence of possible recombination events and of parallel evolutionary changes is shown. Shaded are the proteins characterised in this study. Accession numbers phages: 80 (DQ517338); 53 (AY954952); 11 (AF424781); 85 (AY954953); 69 (AY954951); NM1 (DQ530359); NM2 (DQ530360); ETA2 (AP008953); 12 (AF424782); PVL (AB009866); PVL108 (AB243556); 42E (AY954955); 52A (AY954965); ROSA (AY954961); PVL-CN125 (FJ713816); 77 (AY508486); 47 (AY954957); P954 (GQ398772); NM3 (DQ530361); MR11 (AB370268); 187 (AY954950); SLT (AB045978); tp310-3 (EF462199); 29 (AY954964); 71 (AY954962); 2638A (AY954954); EW (AY954959); 37 (AY954958); PH15 (DQ834250); 13 (AF424783); 55 (AY954963); 80 (DQ908929); MR25 (AB370205). (B). Lineup of the SaPI inducer proteins selected in (A). Adapted from lineup generated by PRALINE. Accession numbers Dut proteins: SLT (NP_075491); 187 (YP_239558); 12 (NP_803328); 2638A (YP_239842); 29 (YP_240597); ROSA (YP_240373); PVL-CN125 (YP_002939700); tp310-3 (YP_001429990). SaPIbov2 inducers: EW (YP_240202); PH15 (YP_950707); 37 (YP_240124); 29 (YP_240582.1); ROSA (YP_240350); MR25 (YP_001949812); ETA2 (YP_001004275). Sri proteins: 52A (YP_240656); 55 (YP_240514); tp310-3 (ERS400827); PVL-CN125 (YP_002939693.1). (PDF) [file pgen.1005609.s003.pdf]

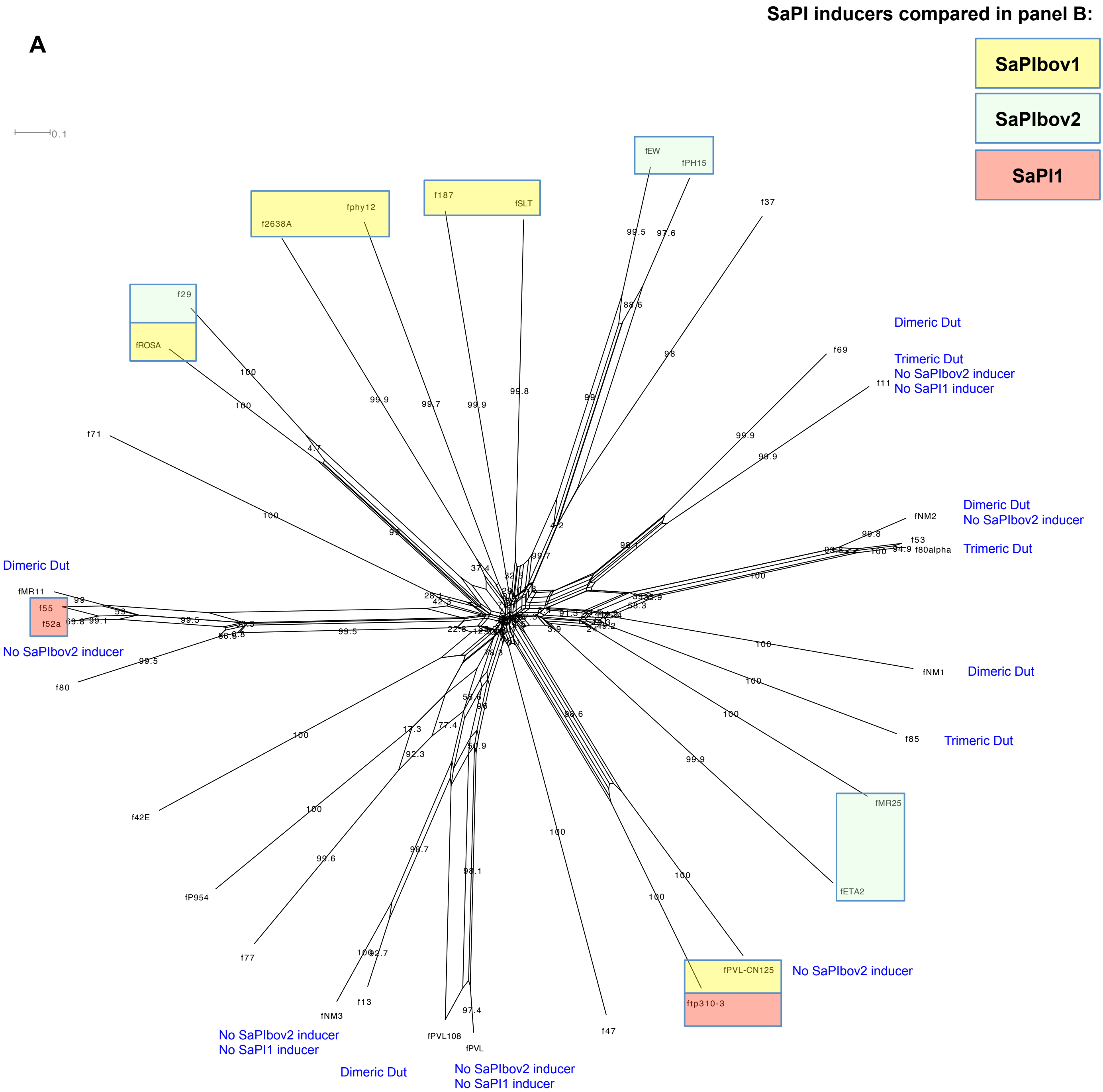

A
SaPI inducers compared in panel B:
SaPIbov1
SaPIbov2
SaPI1
0.1
fEW
fPH15
f187
fSLT
fphy12
f2638A
f29
fROSA
f37
f69
f11
f71
fNM2
f53
f80alpha
fMR11
f55
f52a
f80
fNM1
f85
fMR25
fETA2
f42E
fP954
f77
fNM3
f13
fPVL108
fPVL
f47
fPVL-CN125
ftp310-3
Dimeric Dut
Trimeric Dut
No SaPIbov2 inducer
No SaPI1 inducer

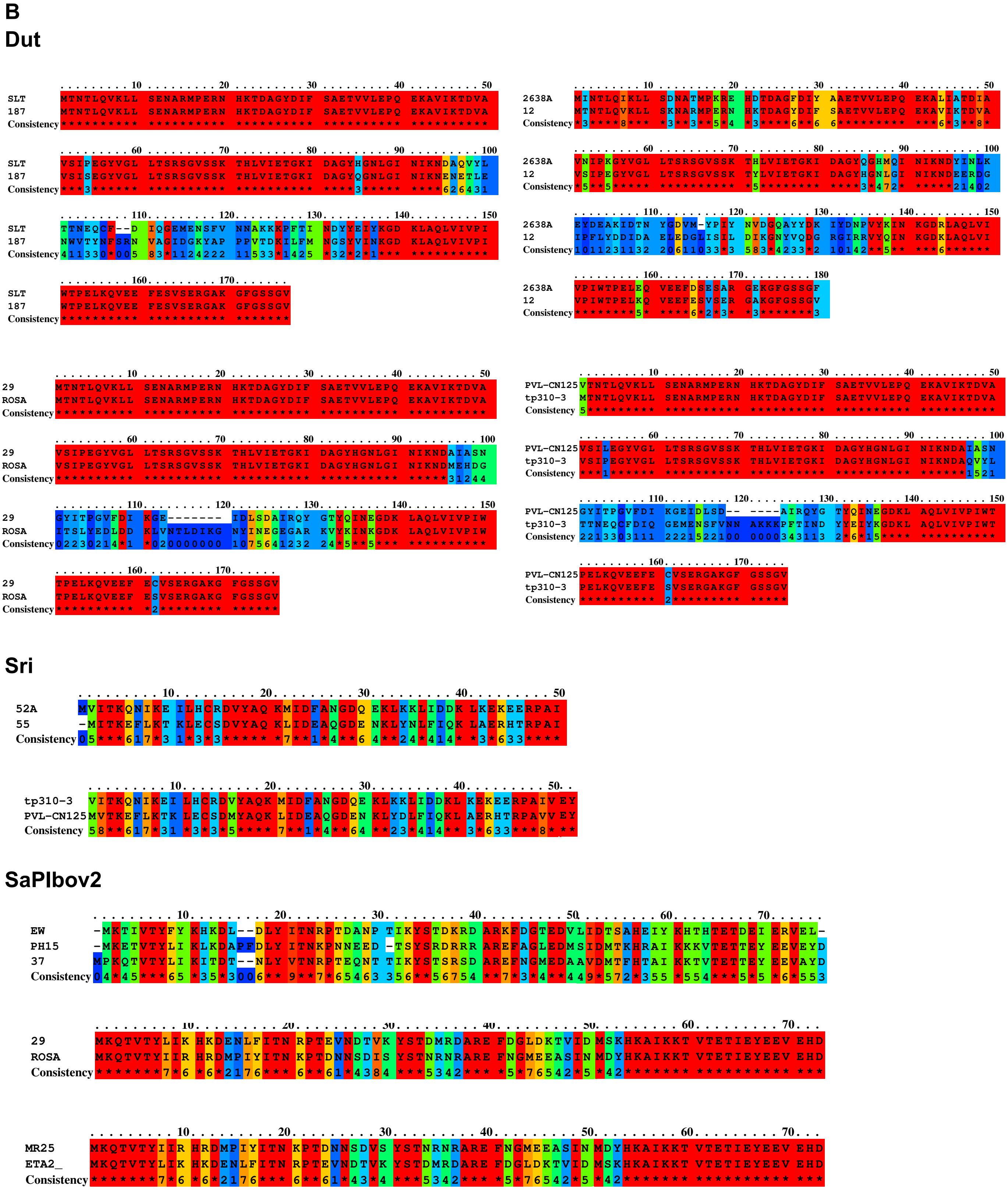
B
Dut
SLT
187
Consistency
2638A
12
29
ROSA
PVL-CN125
tp310-3
Sri
52A
55
SaPIbov2
EW
PH15
37
MR25
ETA2_
