# Supplementary material for: Virus Satellites Drive Viral Evolution and Ecology
Source: PLoS Genet. 2015 Oct 23;11(10):e1005609. doi: 10.1371/journal.pgen.1005609 (PMC4619825; doi:10.1371/journal.pgen.1005609)
Supplement: S1 Table — (PDF) [file pgen.1005609.s006.pdf]

Staphylococcus aureus

Duts[b]	80α	ϕROSA	ϕ71	ϕETA2	ϕPVL108	ϕNM3	ϕ85	ϕ3A	ϕPVL-CN125
80α	0								
ϕROSA	-0,232 (0,076)	0							
ϕ71	-0,161 (0,064)	-0,210 (0,062)	0						
ϕETA2	-0,136 (0,058)	-0,156 (0,059)	-0,113 (0,053)	0					
ϕPVL108	-0,151 (0,063)	-0,178 (0,057)	-0,097 (0,044)	-0,040 (0,044)	0				
ϕNM3	-0,179 (0,073)	-0,218 (0,058)	-0,144 (0,053)	-0,150 (0,054)	-0,113 (0,045)	0			
ϕ85	-0,043 (0,045)	-0,253 (0,065)	-0,141 (0,057)	-0,047 (0,038)	-0,111 (0,049)	-0,153 (0,040)	0		
ϕ3A	-0,269 (0,078)	-0,190 (0,070)	-0,120 (0,055)	-0,130 (0,049)	-0,006 (0,037)	-0,176 (0,063)	-0,192 (0,068)	0	
ϕPVL-CN125	-0,157 (0,064)	-0,206 (0,060)	-0,065 (0,028)	-0,094 (0,051)	-0,061 (0,040)	-0,082 (0,045)	-0,10 (0,051)	-0,116 (0,056)	0

SaPIbov2[c]	ϕROSA	ϕ85	80α	DAR3907	PLAC6004
ϕROSA	0				
ϕ85	-0,011 (0,025)	0			
80α	-0,590 (0,300)	-0,552 (0,282)	0		
DAR3907	-0,631 (0,300)	-0,593 (0,280)	-0,128 (0,064)	0	
PLAC6004	-0,084 (0,054)	-0,059 (0,045)	-0,581 (0,287)	-0,621 (0,282)	0

Sri[d]	A6300	Mu50	ϕ85	21252	M79256	80α	ϕ55
A6300	0						
Mu50	-0,098 (0,069)	0					
ϕ85	-0,089 (0,056)	-0,125 (0,087)	0				
21252	-0,372 (0,190)	-0,363 (0,199)	-0,224 (0,132)	0			
M79256	-1,284 (0,684)	-1,017 (0,627)	-0,740 (0,549)	-0,541 (0,472)	0		
80α	-0,186 (0,094)	-0,090 (0,076)	-0,154 (0,090)	-0,287 (0,161)	-1,148 (0,666)	0	
ϕ55	-0,223 (0,111)	-0,124 (0,089)	-0,256 (0,134)	-0,460 (0,239)	-0,800 (0,587)	-0,136 (0,088)	0

Enterococcus faecalis

E. faecalis[e]	EF0309	Ef11	X98	VC1B-1
EF0309	0			
Ef11	-0,016 (0,346)	0		
X98	0,115 (0,294)	-0,028 (0,029)	0	
VC1B-1	-0,115 (0,493)	-1,085 (0,444)	-1,128 (0,468)	0

[a]The difference between the nonsynonymous and synonymous distances per site from between sequences are shown. Standard error estimate(s) are shown in parenthesis. Analyses were conducted using the modified Nei-Gojobori (assumed transition/transversion bias = 2) model [1]. Evolutionary analyses were conducted in MEGA5 [2].
[b]Accession numbers Dut proteins: 80α (YP_001285346); ϕRosa (YP_240373); ϕ71 (YP_240446); ϕETA2 (YP_001004294); ϕPVL108 (YP_918921); ϕNM3 (YP_908820); ϕ85 (YP_239795); ϕ3A (YP_239989); ϕPVL-CN125 (YP_002939700).
[c]Accession numbers SaPIbov2 inducers: ϕROSA (YP_240350); ϕ85 (YP_239776); 80α (YP_001285329); Strain DAR390 (EXY00034); Strain PLAC6004 (EVF70071).
[d]Accession numbers Sri proteins: Strain A6300 (EEV76885); Strain Mu50 (BAB57032); ϕ85 (YP_239784); Strain 21252 (EHO92899); Strain M79256 (EXP87181); ϕ80α (YP_001285336); ϕ55 (YP_240514).
[e]Accession numbers $EfCI_{V583}$ inducer proteins: EF0309 (AAO80172); Ef11 (YP_003358829); X98 (WP_002381619); VC1B-1(EPI33180).
