# Supplementary material for: Virus Satellites Drive Viral Evolution and Ecology
Source: PLoS Genet. 2015 Oct 23;11(10):e1005609. doi: 10.1371/journal.pgen.1005609 (PMC4619825; doi:10.1371/journal.pgen.1005609)
Supplement: S2 Table — (PDF) [file pgen.1005609.s007.pdf]

Infection SaPIbov1-positive strain

Replica	Sequenced phage	*dut* (SaPIbov1 inducer)	ORF15 (SaPIbov2 inducer)	*sri* (SaPI1 inducer)
1	1	S63I (AGT / ATT)	wt	wt
1	2	S63I (AGT / ATT)	wt	wt
1	3	S63I (AGT / ATT)	wt	wt
1	4	S63I (AGT / ATT)	wt	wt
1	5	A deleted from position 13926	wt	wt
1	6	S63I (AGT / ATT)	wt	wt
1	7	S63I (AGT / ATT)	wt	wt
1	8	S63I (AGT / ATT)	wt	wt
1	9	S63I (AGT / ATT)	wt	wt
1	10	S63I (AGT / ATT)	wt	wt
1	11	S63I (AGT / ATT)	wt	wt
1	12	S63I (AGT / ATT)	wt	wt
1	13	S63I (AGT / ATT)	wt	wt
1	14	S63I (AGT / ATT)	wt	wt
1	15	S63I (AGT / ATT)	wt	wt
1	16	S63I (AGT / ATT)	wt	wt
1	17	S63I (AGT / ATT)	wt	wt
1	18	S63I (AGT / ATT)	wt	wt
1	19	P17S (CCC / TCC)	wt	wt
1	20	S63I (AGT / ATT)	wt	wt
2	1	E155* (GAA / TAA)	wt	wt
2	2	E155* (GAA / TAA)	wt	wt
2	3	E155* (GAA / TAA)	wt	wt
2	4	I75N (ATT / AAT)	wt	wt
2	5	I75N (ATT / AAT)	wt	wt
2	6	I75N (ATT / AAT)	wt	wt
2	7	E155* (GAA / TAA)	wt	wt
2	8	E155* (GAA / TAA)	wt	wt
2	9	E155* (GAA / TAA)	wt	wt
2	10	E155* (GAA / TAA)	wt	wt
2	11	E155* (GAA / TAA)	wt	wt
2	12	E155* (GAA / TAA)	wt	wt
2	13	E155* (GAA / TAA)	wt	wt
2	14	I75N (ATT / AAT)	wt	wt
2	15	E155* (GAA / TAA)	wt	wt
2	16	E155* (GAA / TAA)	wt	wt
2	17	E155* (GAA / TAA)	wt	wt
2	18	E155* (GAA / TAA)	wt	wt
2	19	E155* (GAA / TAA)	wt	wt
2	20	I75N (ATT / AAT)	wt	wt
3	1	I53M (ATA / ATG)	wt	wt
3	2	A25E (GCA / GAA)	wt	wt
3	3	S68I (AGT / ATT)	wt	wt
3	4	E133* (GAA / TAA)	wt	wt
3	5	D24N (GAT / AAT)	wt	wt
3	6	E162* (GAA / TAA)	wt	wt
3	7	R134C (CGT / TGT)	wt	wt
3	8	R15I (AGA / ATA)	wt	wt
3	9	M101K (ATG / AAG)	wt	wt
3	10	A136E (GCA / GAA)	wt	wt
3	11	W144R (TGG / CGG)	wt	wt
3	12	G164D (GGC / GAC)	wt	wt
3	13	W144R (TGG / CGG)	wt	wt
3	14	R15G (AGA / GGA)	wt	wt
3	15	R15I (AGA / ATA)	wt	wt
3	16	G26V (GGT / GTT)	wt	wt
3	17	G59V (GGA / GTA)	wt	wt
3	18	G89E (GGG / GAG)	wt	wt
3	19	G56V (GGC / GTC)	wt	wt
3	20	W144R (TGG / CGG) / V151W (GTG / TGG)	wt	wt

Infection SaPIbov2-positive strain

Replica	Sequenced phage	*dut* (SaPIbov1 inducer)	ORF15 (SaPIbov2 inducer)	*sri* (SaPI1 inducer)
1	1	E133* (GAA / TAA)	T deleted from position 6611	wt
1	2	A deleted from position 14233	A38E (GCA /GAA)	wt
1	3	E133* (GAA / TAA)	T deleted from position 6611	wt
1	4	S63I (AGT / ATT)	T deleted from position 6611	wt
1	5	E133* (GAA / TAA)	T deleted from position 6611	wt
1	6	E133* (GAA / TAA)	T deleted from position 6611	wt
1	7	S63I (AGT / ATT)	A inserted in position 6636	wt
1	8	E133* (GAA / TAA)	T deleted from position 6611	wt
1	9	S63I (AGT / ATT)	A38E (GCA /GAA)	wt
1	10	E133* (GAA / TAA)	T deleted from position 6611	wt
1	11	S63I (AGT / ATT)	A38E (GCA /GAA)	wt
1	12	S63I (AGT / ATT)	S32* (TCA / TAA)	wt
1	13	S63I (AGT / ATT)	A38E (GCA /GAA)	wt
1	14	S63I (AGT / ATT)	S32* (TCA / TAA)	wt
1	15	S63I (AGT / ATT) + A deleted from position 14233	E63* (GAA / TAA)	wt
1	16	E133* (GAA / TAA)	T deleted from position 6611	wt
1	17	S63I (AGT / ATT)	T deleted from position 6611	wt
1	18	S63I (AGT / ATT)	S32* (TCA / TAA)	wt
1	19	S63I (AGT / ATT)	A38E (GCA /GAA)	wt
1	20	S63I (AGT / ATT)	S32P (TCA / CCA)	wt
2	1	I75N (ATT / AAT)	T62P (ACA /CCA)	wt
2	2	I75N (ATT / AAT)	T62P (ACA /CCA)	wt
2	3	I75N (ATT / AAT)	Q3* (CAG / TAG)	wt
2	4	I75N (ATT / AAT)	Q3* (CAG / TAG)	wt
2	5	I75N (ATT / AAT)	T62P (ACA /CCA)	wt
2	6	I75N (ATT / AAT)	T62P (ACA /CCA)	wt
2	7	I75N (ATT / AAT)	T62P (ACA /CCA)	wt
2	8	I75N (ATT / AAT)	Q3* (CAG / TAG)	wt
2	9	I75N (ATT / AAT)	Q3* (CAG / TAG)	wt
2	10	I75N (ATT / AAT)	T62P (ACA /CCA)	wt
2	11	I75N (ATT / AAT)	Q3* (CAG / TAG)	wt
2	12	I75N (ATT / AAT)	T62P (ACA /CCA)	wt
2	13	I75N (ATT / AAT)	T62P (ACA /CCA)	wt
2	14	I75N (ATT / AAT)	Q3* (CAG / TAG)	wt
2	15	I75N (ATT / AAT)	T62P (ACA /CCA)	wt
2	16	I75N (ATT / AAT)	T62P (ACA /CCA)	wt
2	17	I75N (ATT / AAT)	T62P (ACA /CCA)	wt
2	18	I75N (ATT / AAT)	Q3* (CAG / TAG)	wt
2	19	I75N (ATT / AAT)	T62P (ACA /CCA)	wt
2	20	I75N (ATT / AAT)	T62P (ACA /CCA)	wt
3	1	M101K (ATG / AAG)	A38E (GCA / GAA)	wt
3	2	M101K (ATG / AAG)	A38E (GCA / GAA)	wt
3	3	A inserted in position 14383	E24* (GAA / TAA)	wt
3	4	W144R (TGG / CGG)	A38E (GCA / GAA)	wt
3	5	R15I R15I (AGA / ATA)	E40* (GAA / TAA)	wt
3	6	M101K (ATG / AAG)	A38E (GCA / GAA)	wt
3	7	E112K (GAA / AAA)	S32P (TCA / CCA)	wt
3	8	C deleted from position 14054	wt	wt
3	9	M101K (ATG / AAG)	A38E (GCA / GAA)	wt
3	10	M101K (ATG / AAG)	A38E (GCA / GAA)	wt
3	11	R15I (AGA / ATA)	E40* (GAA / TAA)	wt
3	12	R15I (AGA / ATA)	A38E (GCA / GAA)	wt
3	13	I143T (ATA / ACA)	S32* (TCA / TAA)	wt
3	14	R15I (AGA / ATA)	E40* (GAA / TAA)	wt
3	15	K79N (AAG / AAT)	A38E (GCA / GAA)	wt
3	16	I53T (ATA / ACA)	A38E (GCA / GAA)	wt
3	17	A50V (GCT / GTT)	A38E (GCA / GAA)	wt
3	18	G59R (GGA / CGA)	E40* (GAA / TAA)	wt
3	19	E130K (GAA / AAA)	E40* (GAA / TAA)	wt
3	20	A50V (GCT / GTT)	T62P (ACA /CCA)	wt

Infection SaPI1-positive strain

Replica	Sequenced phage	*dut* (SaPIbov1 inducer)	ORF15 (SaPIbov2 inducer)	*sri* (SaPI1 inducer)
1	1	S63I (AGT / ATT)	E63* (GAA / TAA)	Q19* (CAG / TAG)
1	2	E133* (GAA / TAA)	T deleted from position 6611	Y17H (TAC / CAC)
1	3	E133* (GAA / TAA)	T deleted from position 6611	A18D (GCT / GAT)
1	4	E133* (GAA / TAA)	T deleted from position 6611	Y17H (TAC / CAC)
1	5	E133* (GAA / TAA)	wt	G 10983A (ribosoma binding site)
1	6	S63I (AGT / ATT)	A inserted in position 6636	L7S (TTG / TCG)
1	7	S63I (AGT / ATT)	S32P (TCA / CCA)	Y17H (TAC / CAC)
1	8	P39L (CCA / CTA)	T deleted from position 6611	S14L (TCA / TTA)
1	9	S63I (AGT / ATT)	Q3* (CAG / TAG)	C13R (TGT / CGT)
1	10	E133* (GAA / TAA)	T deleted from position 6611	A25E (GCA / GAA)
1	11	E133* (GAA / TAA)	T deleted from position 6611	T deleted from position 11011
1	12	S63I (AGT / ATT)	T deleted from position 6611	S14* (TCA / TAA)
1	13	P39L (CCA / CTA)	wt	Y17H (TAC / CAC)
1	14	S63I (AGT / ATT)	A inserted in position 6636	A25E (GCA / GAA)
1	15	E133* (GAA / TAA)	A38E (GCA /GAA)	S14* (TCA / TAA)
1	16	P39L (CCA / CTA)	wt	Y17H (TAC / CAC)
1	17	P39L (CCA / CTA)	wt	Y17H (TAC / CAC)
1	18	wt	S32P (TCA / CCA)	Y17H (TAC / CAC)
1	19	A32T (GCT / ACT)	wt	A18T (GCT / ACT)
1	20	S63I (AGT / ATT)	A38E (GCA /GAA)	G 10983 A (ribosoma binding site)
2	1	I75N (ATT / AAT)	Q3* (CAG / TAG)	E24* (GAG / TAG)
2	2	E55* (GAA / TAA)	A38E (GCA / GAA)	C13F (TGT /TTT)
2	3	I75N (ATT / AAT)	Q3* (CAG / TAG)	E24* (GAG / TAG)
2	4	I75N (ATT / AAT)	Q3* (CAG / TAG)	several deletions
2	5	I75N (ATT / AAT)	T62P (ACA /CCA)	T deleted from position 11040
2	6	I75N (ATT / AAT)	T62P (ACA /CCA)	A18D (GCT / GCA)
2	7	I75N (ATT / AAT)	T62P (ACA /CCA)	G 10983 A (ribosoma binding site)
2	8	I75N (ATT / AAT)	Q3* (CAG / TAG)	Y17H (TAC / CAC)
2	9	I75N (ATT / AAT)	Q3* (CAG / TAG)	E24* (GAG / TAG)
2	10	I75N (ATT / AAT)	Q3* (CAG / TAG)	E24* (GAG / TAG)
2	11	I75N (ATT / AAT)	T62P (ACA /CCA)	G 10983 A (ribosoma binding site)
2	12	I75N (ATT / AAT)	Q3* (CAG / TAG)	E24* (GAG / TAG)
2	13	I75N (ATT / AAT)	T62P (ACA /CCA)	G 10983 A (ribosoma binding site)
2	14	I75N (ATT / AAT)	Q3* (CAG / TAG)	E24* (GAG / TAG)
2	15	I75N (ATT / AAT)	T62P (ACA /CCA)	G 10983 A (ribosoma binding site)
2	16	I75N (ATT / AAT)	T62P (ACA /CCA)	G 10983 A (ribosoma binding site)
2	17	I75N (ATT / AAT)	Q3* (CAG / TAG)	E24* (GAG / TAG)
2	18	I75N (ATT / AAT)	T62P (ACA /CCA)	G 10983 A (ribosoma binding site)
2	19	I75N (ATT / AAT)	T62P (ACA /CCA)	G 10983 A (ribosoma binding site)
2	20	I75N (ATT / AAT)	Q3* (CAG / TAG)	E24* (GAG / TAG)
3	1	A inserted in position 13931	S32P (TCA / CCA)	E24* (GAG / TAG)
3	2	A inserted in position 13931	S32P (TCA / CCA)	E24*(GAG / TAG)
3	3	A inserted in position 13931, AGA insertion in 14195	A38E (GCA / GAA)	Y17H (TAC / CAC)
3	4	R15I (AGA / ATA)	A38E (GCA / GAA)	A deleted from 11062
3	5	G164S (GGC / AGC)	A38E (GCA / GAA)	C13Y (TGT / TAT)
3	6	I45S (ATC / AGC)	S32P (TCA / CCA)	C13Y (TGT / TAT)
3	7	A32D (GCT / GAT) and E118* (GAA / TAA)	E63* (GAA / TAA)	G 10983 A (ribosome binding site)
3	8	M101K (ATG / AAG) and G inserted in position 14096	A38E (GCA / GAA)	A18D (GCT / GAT)
3	9	I53T (ATA / ACA)	A38E (GCA / GAA)	L7S (TTG / TCG)
3	10	R15I (AGA / ATA)	A38E (GCA / GAA)	A deleted from 11062
3	11	V51L (GTG / TTG)	30DEL61	G deleted from 11025
3	12	I53T (ATA / ACA)	A38E (GCA / GAA)	T deleted from 11040
3	13	wt	A38E (GCA / GAA)	C13Y (TGT / TAT)
3	14	G164S (GGC / AGC)	E40* (GAA / TAA)	C13Y (TGT / TAT)
3	15	wt	A38E (GCA / GAA)	C13Y (TGT / TAT)
3	16	I45S (ATC / AGC)	S32P (TCA / CCA) and E40* (GAA / TAA)	C13Y (TGT / TAT)
3	17	133DEL164	A38E (GCA / GAA)	Y17H (TAC / CAC)
3	18	133DEL164	A38E (GCA / GAA)	Y17H (TAC / CAC)
3	19	M101K (ATG / AAG)	A38E (GCA / GAA)	A18D (GCT / GAT)
3	20	133DEL164	S32P (TCA / CCA)	C13Y (TGT / TAT)
