# Supplementary material for: Virus Satellites Drive Viral Evolution and Ecology
Source: PLoS Genet. 2015 Oct 23;11(10):e1005609. doi: 10.1371/journal.pgen.1005609 (PMC4619825; doi:10.1371/journal.pgen.1005609)
Supplement: S3 Table — (PDF) [file pgen.1005609.s008.pdf]

80α genotype	SaPI	Phage titre[b]	SaPI titre[c]
wt	-	3.1×10^{10}	-
wt	SaPIbov1	4.6×10^{8}	5.2×10^{7}
wt	SaPIbov2	3.4×10^{7}	1.1×10^{8}
wt	SaPI1	1.5×10^{8}	1.7×10^{8}
Dut I75N; ORF15 T62P; Sri G10983A	-	9.3×10^{9}	-
Dut I75N; ORF15 T62P; Sri G10983A	SaPIbov1	8.6×10^{9}	4.7×10^{2}[d]
Dut I75N; ORF15 T62P; Sri G10983A	SaPIbov2	9.4×10^{9}	9.7×10^{2}[d]
Dut I75N; ORF15 T62P; Sri G10983A	SaPI1	1.1×10^{10}	1.7×10^{2}[d]
Dut G164S; ORF15 E40*; Sri C13Y	-	2.1×10^{10}	-
Dut G164S; ORF15 E40*; Sri C13Y	SaPIbov1	2.6×10^{10}	4.4×10^{2}[d]
Dut G164S; ORF15 E40*; Sri C13Y	SaPIbov2	1.1×10^{10}	1.4×10^{3}[d]
Dut G164S; ORF15 E40*; Sri C13Y	SaPI1	1.3×10^{10}	1.1×10^{2}[d]

[a]The means of results from three independent experiments are presented. Variation was within 5% in all cases.
[b]Plaques / ml of lysate, using RN4220 as indicator.
[c]Transductants / ml of lysate, using RN4220 as recipient.
[d]This frequency is typical of transfer by generalized transduction and is not SaPI-specific.
