# Supplementary material for: Virus Satellites Drive Viral Evolution and Ecology
Source: PLoS Genet. 2015 Oct 23;11(10):e1005609. doi: 10.1371/journal.pgen.1005609 (PMC4619825; doi:10.1371/journal.pgen.1005609)
Supplement: S4 Table — (PDF) [file pgen.1005609.s009.pdf]

Inducing Phage 80α	SaPI	Branch	SaPI mutation[a,b]
Dut S63I, ORF15 A38E, *sri* G 10983 A	SaPIbov1	1	A deleted from position 14611. Stl mutant.
Dut S63I, ORF15 A38E, *sri* G 10983 A	SaPIbov1	2	A deleted from position 14119. Stl mutant.
Dut S63I, ORF15 A38E, *sri* G 10983 A	SaPIbov1	3	Stl M7I.
Dut S63I, ORF15 A38E, *sri* G 10983 A	SaPIbov1	4	A deleted from position 14303. Stl mutant.
Dut S63I, ORF15 A38E, *sri* G 10983 A	SaPIbov2	1	Deletion in *stl*. Residues N122-K168 eliminated.
Dut S63I, ORF15 A38E, *sri* G 10983 A	SaPIbov2	2	Stl G110R.
Dut S63I, ORF15 A38E, *sri* G 10983 A	SaPIbov2	3	C-terminal mutation. The last K* is substituted by PES*.
Dut I75N, ORF15 Q3*, Δ*sri*	SaPIbov1	1	A deleted from position 14119. Stl mutant.
Dut I75N, ORF15 Q3*, Δ*sri*	SaPIbov1	2	Stl Q150*. Stl mutant.
Dut I75N, ORF15 Q3*, Δ*sri*	SaPIbov1	3	T inserted in position 14373. Stl mutant.
Dut I75N, ORF15 Q3*, Δ*sri*	SaPIbov1	4	A inserted in position 14454. Stl mutant.
Dut I75N, ORF15 Q3*, Δ*sri*	SaPIbov2	1	T deleted from position 23963 (*stl* promoter region).
Dut I75N, ORF15 Q3*, Δ*sri*	SaPIbov2	2	C-terminal mutation. The last EIDKYLK* residues are substituted by AQSKL*
wt	SaPIbov1	1	No mutations.
wt	SaPIbov1	2	No mutations.
wt	SaPIbov1	3	No mutations.
wt	SaPIbov1	4	No mutations.
wt	SaPIbov2	1	No mutations.
wt	SaPIbov2	2	No mutations.
wt	SaPIbov2	3	No mutations.
wt	SaPIbov2	4	No mutations.

[a]GenBank accession number for SaPIbov1: AF217235.
[b]GenBank accession number for SaPIbov2: AY220730.
