# Supplementary material for: Virus Satellites Drive Viral Evolution and Ecology
Source: PLoS Genet. 2015 Oct 23;11(10):e1005609. doi: 10.1371/journal.pgen.1005609 (PMC4619825; doi:10.1371/journal.pgen.1005609)
Supplement: S5 Table — (PDF) [file pgen.1005609.s010.pdf]

Sequenced phage	Xis mutation
1	M1R
2	W43*
3	M1*
