# Supplementary material for: Virus Satellites Drive Viral Evolution and Ecology
Source: PLoS Genet. 2015 Oct 23;11(10):e1005609. doi: 10.1371/journal.pgen.1005609 (PMC4619825; doi:10.1371/journal.pgen.1005609)
Supplement: S6 Table — (PDF) [file pgen.1005609.s011.pdf]

Strains	Description	Reference
RN4220	*Staphylococcus aureus*. Restriction-defective derivative of RN450	Lab strain
RN10359	RN450 lysogenic for 80α	Lab strain
JP10977	*Enterococcus faecalis* V583 cured of its plasmids	[3]
JP10983	ϕ1, ϕ3, ϕ4, ϕ5, ϕ6 and ϕ7 deletion in JP10977	[3]
JP10982	JP10983 EfCIV583-positive	[3]
JP10984	JP10983 lysogenic for ϕ1	[3]
JP1996	RN4220 SaPIbov1 *tst::tet*M	[4]
JP2129	RN4220 SaPIbov2 *bap::tet*M	[5]
JP2966	RN4220 SaPI1 *tst::tet*M	[4]
JP3603	RN10359 SaPIbov1 *tst::tet*M	[6]
JP6764	RN10359 SaPIbov2 *tst::tet*M	[6]
JP7582	RN4220 ϕ80α Dut E55*; ORF15 A38E; Sri C13F	This study
JP7587	RN4220 ϕ80α Dut I75N; ORF15 T62P; Sri G10983A	This study
JP8616	RN4220 ϕ80α Dut G164S; ORF15 E40*; Sri C13Y	This study
JP8615	RN4220 ϕ80α Dut A insertion 13931; ORF15 S32P; Sri E24*	This study
JP8361	RN4220 ϕ55-2	This study
JP8362	RN4220 ϕ55-3	This study
JP7583	RN4220 ϕ80α Dut I75N; ORF15 Q3*; Δ*sri*	This study
JP9354	JP7583 SaPIbov1 *tst::tet*M	This study
JP12774	JP7583 SaPIbov2 *tst::tet*M	This study
JP11389	RN4220 ϕ80α Dut S63I; ORF15 A38E; Sri G10983A	This study
JP12767	JP11389 SaPIbov1 *tst::tet*M	This study
JP12768	JP11389 SaPIbov2 *tst::tet*M	This study
JP12829	JP11389 SaPIbov1 *tst::tet*M A deleted from position 14611	This study
JP12929	JP11389 SaPIbov1 *tst::tet*M A deleted from position 14119	This study
JP12930	JP11389 SaPIbov1 *tst::tet*M Stl M7I	This study
JP12931	JP11389 SaPIbov1 *tst::tet*M A deleted from position 14303	This study
JP12830	JP11389 SaPIbov2 *tst::tet*M Δ*stl.* Residues N122-K168 eliminated	This study
JP12953	JP11389 SaPIbov2 *tst::tet*M Stl G110R	This study
JP12954	JP11389 SaPIbov2 *tst::tet*M Stl carrying a C-terminal mutation. The last K* is substituted by PES*.	This study
JP12831	JP7583 SaPIbov1 *tst::tet*M A deleted from position 14119	This study
JP12932	JP7583 SaPIbov1 *tst::tet*M Stl Q150*	This study

Strains	Description	Reference
JP12933	JP7583 SaPIbov1 *tst::tet*M T inserted in position 14373	This study
JP12934	JP7583 SaPIbov1 *tst::tet*M A inserted in position 14454	This study
JP13000	JP7583 SaPIbov2 *tst::tet*M T deleted from position 23963	This study
JP12956	JP7583 SaPIbov2 *tst::tet*M Stl C-terminal mutation. The last EIDKYLK* residues are substituted by AQSKL*	This study
JP12848	RN4220 SaPIbov1 *tst::tet*M A deleted from position 14303	This study
JP12846	RN4220 SaPIbov2 *tst::tet*M Δ*stl.* Residues N122-K168 eliminated	This study
JP8059	RN450 Δ*spa* SaPIbov1 *tst*::*tet*M	This study
JP8116	JP8059 pCN51-3xflag-*dut* ϕ11 (with RBS from phage 80α)	This study
JP8117	JP8059 pCN51-3xflag-*dut* 80α	This study
JP8691	JP8059 pCN51-3xflag-*dut* 80α G164S	This study
JP8938	JP8059 pCN51-3xflag-*dut* 80α I75N	This study
JP8940	JP8059 pCN51-3xflag-*dut* 80α A32T	This study
JP10376	JP8059 pCN51-3xflag-*dut* B2 (with RBS from phage 80α)	This study
JP10531	JP8059 pCN51-3xflag-*dut* ϕSaov3 (with RBS from phage 80α)	This study
BL21(DE3)	*E. coli* expression strain	Stratagene
JP10635	BL21(DE3) pET28a-*dut* 80α I75N	This study
JP10636	BL21(DE3) pET28a-*dut* 80α G164S	This study
JP10637	BL21(DE3) pET28a-*dut* 80α A32T	This study
JP10641	BL21(DE3) pET28a-*dut* B2	This study

[a]GenBank accession number for SaPIbov1: AF217235.
[b]GenBank accession number for SaPIbov2: AY220730.
