# Supplementary material for: Virus Satellites Drive Viral Evolution and Ecology
Source: PLoS Genet. 2015 Oct 23;11(10):e1005609. doi: 10.1371/journal.pgen.1005609 (PMC4619825; doi:10.1371/journal.pgen.1005609)
Supplement: S7 Table — (PDF) [file pgen.1005609.s012.pdf]

Plasmid	Description	Reference
pCN51	Expression vector	[7]
pET-28a	Expression vector	Novagen
pJP821	pCN51-3xflag-*dut* 80α	[6]
pJP1111	pCN51-3xflag-*dut* 80α I75N	This study
pJP1113	pCN51-3xflag-*dut* 80α A32T	This study
pJP1553	pCN51-3xflag-*dut* 80α G164S	This study
pJP813	pCN51-3xflag-*dut* ϕ11 (RBS 80α)	This study
pJP1657	pCN51-3xflag-*dut* ϕSaov3 (RBS 80α)	This study
pJP1658	pCN51-3xflag-*dut* B2 (RBS 80α)	This study
pJP1655	pET28*a*-*dut* ϕ80α I75N	This study
pJP1562	pET28*a*-*dut* ϕ80α G164S	This study
pJP1563	pET28*a*-*dut* ϕ80α A32T	This study
pJP1656	pET28*a*-*dut* B2	This study
