# Supplementary material for: Virus Satellites Drive Viral Evolution and Ecology
Source: PLoS Genet. 2015 Oct 23;11(10):e1005609. doi: 10.1371/journal.pgen.1005609 (PMC4619825; doi:10.1371/journal.pgen.1005609)
Supplement: S8 Table — (PDF) [file pgen.1005609.s013.pdf]

Plasmid	Oligonucleotides	Sequence (5'-3')
pJP821 pJP1111 pJP1113	Orf-32-phi80alpha-18mS	ACGCGTCGACATTATGGCAGGTCAAGTTGTCTATAAATATGAGGAGGAATAGGAAAATGGATTATAAAGATCACGATGG
	Orf-32-phi80alpha-17m	ATGGATTATAAAGATCACGATGGCGATTATAAAGATCACGATATCGATTATAAAGATGATGATGATAAAATGACTAACACATTACAAGTAAAAC
	Orf32phi80alpha-2cB	CGCGGATCCTCACCAAAACCTCCTTGACTC
pJP813	Orf-32-phi80alpha-18mS	ACGCGTCGACATTATGGCAGGTCAAGTTGTCTATAAATATGAGGAGGAATAGGAAAATGGATTATAAAGATCACGATGG
	orf25-phi11-35m	ATGGATTATAAAGATCACGATGGCGATTATAAAGATCACGATATCGATTATAAAGATGATGATGATAAAATGACTAACACATTACAAGTAAGG
	Orf25phi11-5cB	CGCGGATCCCTTGACTCGATCTAAGATGTC
pJP1657	Orf-32-phi80alpha18mS	ACGCGTCGACATTATGGCAGGTCAAGTTGTCTATAAATATGAGGAGGAATAGGAAAATGGATTATAAAGATCACGATGG
	dut-ED133-3xflag-1m	ATGGATTATAAAGATCACGATGGCGATTATAAAGATCACGATATCGATTATAAAGATGATGATGATAAAATGACTAACATCCTACAAGTG
	dut-ED133-2cB	CGCGGATCCCTTGACCTTATCTAATATGTC
pJP1658	Orf-32-phi80alpha18mS	ACGCGTCGACATTATGGCAGGTCAAGTTGTCTATAAATATGAGGAGGAATAGGAAAATGGATTATAAAGATCACGATGG
	Orf-32-phi80alpha-17m	ATGGATTATAAAGATCACGATGGCGATTATAAAGATCACGATATCGATTATAAAGATGATGATGATAAAATGACTAACACATTACAAGTAAAAC
	dut-BII-1cB	CGCGGATCCTAACACTCCGCTACTTCCGAAGCCTTTTTC
pJP1562 pJP1563 pJP1655 pJP1656	Orf32-phi80alpha-12mB	CGCGGATCCATGACTAACACATTACAAGTAAAAC
	Orf32-phi80alpha-13cS	ACGCGTCGACTCTTTACACTCCGCTACTTCC

*Underlined is shown the sequence recognized by the restriction enzymes used.

SaPI inducers (PCR amplification)	Oligonucleotides	Sequence (5'-3')
dut 80α	Orf32-phi80α-1mS	ACGCGTCGACGAACTTGATGAAACAGTAGGG
	Orf32- phi80α-2cB	CGCGGATCCTCACCAAAACCTCCTTGACTC
ORF15 80α	Orf15-phi80α-7mB	CGCGGATCCTAATTAACTTAGTCATGAGATGG
	Orf15- phi80α-8cK	CGGGGTACCTTCAGTCATGTTCTACCTCC
sri 80α	Orf22-phi80α-4mB	CGCGGATCCAAGTAAGAGTAATCGGAGACG
	Orf22- phi80α-5cE	CCGGAATTCCTTTCGGCAATTTTTAACACTCC
sri ϕ55-2	Orf103phi55-8m	GATACGATCATAACTATTTGG
	Orf103phi55-9c	GCAAGTTTTTGGATAAATAGG
sri ϕ55-3	Orf103phi55-6m	TGAGGATTCTAAAGGTTTTCC
	Orf103phi55-7c	TTTCTCTTGGTCTCCGTTTGC
Flanking divergent region ϕ55-2ϕ / 55-3	Orf103phi55-5m	ACATCATAACCAGCAACATGC
	Orf103-phi55-1c	CTTAATATTCGACGATAGCGG

Southern blot	Oligonucleotides	Sequence (5’-3’)
SaPIbov1 probe	SaPIbov1-112mE	CCGGAATTCAATTGCTGAGGCAAAACTTC
	SaPIbov1-113cB	CGCGGATCCTAATTCTCCACGTCTAAAGC

References

1. Zhang J, Rosenberg HF, Nei M (1998) Positive Darwinian selection after gene duplication in primate ribonuclease genes. Proc Natl Acad Sci USA 95: 3708–3713.

2. Tamura K, Peterson D, Peterson N, Stecher G, Nei M, et al. (2011) MEGA5: molecular evolutionary genetics analysis using maximum likelihood, evolutionary distance, and maximum parsimony methods. Mol Biol Evol 28: 2731–2739.

3. Matos RC, Lapaque N, Rigottier-Gois L, Debarbieux L, Meylheuc T, et al. (2013) *Enterococcus faecalis* prophage dynamics and contributions to pathogenic traits. PLoS Genet 9: e1003539.

4. Ubeda C, Maiques E, Barry P, Matthews A, Tormo MA, et al. (2008) SaPI mutations affecting replication and transfer and enabling autonomous replication in the absence of helper phage. Mol Microbiol 67: 493–503. d

5. Maiques E, Ubeda C, Tormo MA, Ferrer MD, Lasa I, et al. (2007) Role of staphylococcal phage and SaPI integrase in intra- and interspecies SaPI transfer. J Bacteriol 189: 5608–5616.

6. Tormo-Más MÁ, Mir I, Shrestha A, Tallent SM, Campoy S, et al. (2010) Moonlighting bacteriophage proteins derepress staphylococcal pathogenicity islands. Nature 465: 779–782.

7. Charpentier E, Anton AI, Barry P, Alfonso B, Fang Y, et al. (2004) Novel cassette-based shuttle vector system for gram-positive bacteria. Appl Environ Microbiol 70: 6076–6085.
